# Review of the Neotropical genus *Rhyncholepta* with descriptions of three new species-group taxa (Hemiptera, Heteroptera, Pentatomidae)

**DOI:** 10.3897/zookeys.796.22517

**Published:** 2018-11-15

**Authors:** Petr Kment, Joe E. Eger, Jr. David A. Rider3

**Affiliations:** 1 Department of Entomology, National Museum, Cirkusová 1740, 19300 Prague 9, Czech Republic Department of Entomology, National Museum Prague Czech Republic; 2 Florida State Collection of Arthropods, 2606 S. Dundee St., 33629 Tampa, FL, USA Florida State Collection of Arthropods Tampa United States of America; 3 Department of Entomology, North Dakota State University, Fargo, North Dakota, USA North Dakota State University Fargo United States of America

**Keywords:** distribution, Hemiptera, Heteroptera, key, Neotropical Region, new species, new subspecies, new record, Pentatomidae, phenology, taxonomy

## Abstract

The genus *Rhyncholepta* Bergroth, 1911 (Hemiptera: Heteroptera: Pentatomidae: Pentatominae: Chlorocorini) is redescribed and five species-group taxa are recognized, keyed, their diagnostic characters illustrated, and the distribution reviewed. Among the five taxa, two species and one subspecies are recognized as new: *Rhyncholeptagrandicallosagrandicallosa* Bergroth, 1911 (Brazil, Ecuador, French Guiana, Guyana, Peru, Suriname), *Rhyncholeptagrandicallosacentroamericana***subsp. n.** (Belize, Costa Rica, Guatemala, Mexico, Panama), *Rhyncholeptahenryi***sp. n.** (French Guiana), *Rhyncholeptameinanderi* Becker & Grazia-Vieira, 1971 (Bolivia, Brazil, Ecuador, Peru), and *Rhyncholeptawheeleri***sp. n.** (Guyana). The structure of the male genital capsule was found to be the only reliable character for identifying species-group taxa. For this reason, a simultaneous application has been submitted to the International Commission on Zoological Nomenclature to set aside the non-informative female lectotype of *Rhyncholeptagrandicallosagrandicallosa* and replace it with the male neotype suggested herein. Based on the available label data and our field experience, most of the specimens were collected by various types of light traps in or near dense forests. Adults can be collected throughout the year.

## Introduction

Bergroth (1911) described a new pentatomid genus, *Rhyncholepta* Bergroth, 1911, which included the new species *Rhyncholeptagrandicallosa* Bergroth, 1911, from French Guiana. He originally placed *Rhyncholepta* in the subfamily Arminae (currently valid name Asopinae) because of a habitus superficially similar to that of members of the asopine genera *Apateticus* Dallas, 1851 and *Podisus* Herrich-Schaeffer, 1851, and also listed several structural differences including the eponymous slender rostrum (Bergroth 1911, 1914), which actually excludes it from that subfamily. Bergroth (1914) provided an excellent color painting of *Rh.grandicallosa*. The genus *Rhyncholepta* was not mentioned during the following decades until Pirán (1956), who reported a male from Bolivia, provided a line drawing of the abdominal apex and the genital capsule in ventral view, and erroneously designated it as the allotype of *Rh.grandicallosa*. Becker and Grazia-Vieira (1971) first studied this genus in detail including descriptions of the external and internal genitalia of both sexes and distinguished two species, *Rh.grandicallosa* (interpretation based on examination of the female primary type) and a new species, *Rh.meinanderi* Becker & Grazia-Vieira, 1971 (= *Rh.grandicallosa* sensu Pirán). They transferred *Rhyncholepta* from the Asopinae to the Pentatominae, tribe Pentatomini, indicating that the genus is probably related to *Loxa* Amyot & Serville, 1843, and *Fecelia* Stål, 1872 (cf. Thomas 1992). Despite the transfer of *Rhyncholepta* to the Pentatominae, the genus was omitted in keys to the pentatomine genera of the New World (cf. Rolston and McDonald 1981, 1984; Rolston et al 1980). *Rhyncholepta* since has been included in a generic key by Torres Gutiérrez (2005), and several authors have provided new distributional records or checklists from Honduras, Panama, Colombia, Venezuela, Brazil and Peru (Grazia 1984, Froeschner 1999, Arismendi and Thomas 2003, Arnold 2011, Castro-Huertas et al. 2015, Cambra et al. 2018, Rider et al. 2018, Silva et al. 2018). Greve et al. (2013) included *Rh.grandicallosa*, along with *Loxadeducta* Walker, 1867 and *Mayriniacurvidens* (Mayr, 1864), as out-groups in their cladistic analysis of *Chloropepla* Stål, 1867, considering them closely related (based mainly on the presence of the hypandrium on the ventral wall of the genital capsule). The hypothesized relationship of *Rhyncholepta* with the allied genera was formalized by the description of the tribe Chlorocorini Rider, Greve, Schwertner & Grazia, 2018 (in Rider et al. 2018), including *Arvelius* Spinola, 1837, *Chlorocoris* Spinola, 1837 (with 3 subgenera: *Arawacoris* Thomas, 1998, *Chlorocoris*, and *Monochrocerus* Stål, 1872), *Chloropepla*, *Eludocoris* Thomas, 1992, *Fecelia*, *Loxa*, and *Mayrinia* Horváth, 1925.

Our examination of representative material of 1125 specimens of *Rhyncholepta* collected in the last few decades revealed a very complicated situation in this genus. We distinguish five species-group taxa based on the structure of the genital capsule of the male. The existence of two morphologically nearly identical species syntopic in the area of French Guiana (type locality of *Rh.grandicallosa*) required reinterpretation of the taxon *Rh.grandicallosa*. Females, however, are insufficiently informative to serve this purpose; therefore, we selected a male candidate neotype for *Rh.grandicallosagrandicallosa* Bergroth, 1911, and we are asking the International Commission on Zoological Nomenclature to set aside the non-informative female lectotype (Kment et al., submitted).

## Materials and methods

Labels of the name-bearing types are quoted verbatim. A slash (/) is used to divide data on different lines of one label, a double slash (//) to divide data on different labels, and authors’ comments are given in square brackets []. Label data of paratypes and other non-type material are provided in standardized format.

The following dimensions were measured: total body length (from apex of mandibular plates to apex of membrane, in dorsal view), body length to segment VII (from apex of mandibular plates to apices of segment VII, in dorsal view), head length (from apex of mandibular plates to anterior margin of pronotum, in anterodorsal view, i.e. with surface of the head parallel with the plane of focus), head width (maximum width across eyes, in anterodorsal view), interocular width (between inner margins of compound eyes, in anterodorsal view), length of each antennal segment (maximum length), pronotum length (medially, in most exposed – i.e., anterodorsal – view), pronotum width (maximum width including humeral processes, in dorsal view), scutellum length (medially from base to apex, in dorsal view), and scutellum width (maximum width at base, in dorsal view). Measurements are presented, in millimeters, as median and minimum-maximum range.

Uncoated specimens were examined by a Hitachi S-3700N environmental scanning electron microscope at the Department of Palaeontology, National Museum, Prague. Habitus photographs were taken in National Museum in Prague using a Canon MP-E 65 mm macro lens attached to a Canon EOS 550D camera and stacked from multiple layers using the Helicon Focus 5.1 Pro software. Photos by Joe Eger were taken and edited using Auto-Montage™ software (Syncroscopy, Cambridge, UK) at the Florida State Collection of Arthropods, Gainesville, FL.

Morphological terminology mostly follows Tsai et al. (2011), Tsai and Rédei (2014), Kment (2015), Kment et al. (2016) and Rédei (2017). Parts of the thoracic efferent system of the metathoracic scent glands are named in accordance with Kment and Vilímová (2010). The nomenclature of antennomeres follows Zrzavý (1990): scape (I), pedicel subdivided into basipedicellite (IIa) and distipedicellite (IIb), basiflagellum (III) and distiflagellum (IV).

The distribution maps were processed in QGIS 2.18 (qgis.org/en/site/forusers/download.html) using the geographic co-ordinates provided on labels or acquired subsequently using Google; the latter are given in square brackets.

The material examined or cited is deposited in the following collections:

**BMNH** The Natural History Museum, London, United Kingdom

**DARC** David A. Rider collection, Fargo, North Dakota, USA

**DBTC** Donald B. Thomas collection, Edinburgh, Texas, USA

**FSCA** Florida State Collection of Arthropods, Gainesville, Florida, USA

**HNHM** Hungarian Natural History Museum, Budapest, Hungary

**INBIO** Instituto Nacional de Biodiversidad, San José, Costa Rica

**INPA** Instituto Nacional de Pesquisas da Amazonia, Manaus, Amazonas, Brazil

**IZAV** Instituto de Zoologia Agricola, Universidad Central de Venezuela, Maracay, Venezuela

**JEEC** Joe E. Eger collection, Tampa, Florida, USA

**MMBC** Moravian Museum, Brno, Czech Republic

**MNHN** Muséum national d’Histoire naturelle, Paris, France

**MZHF** Muséum national d’Histoire naturelle, Paris, France

**NHMW** Naturhistorisches Museum in Wien, Vienna, Austria

**NMPC** National Museum, Prague, Czech Republic

**RLFF** Roland Lupoli collection, Fontenay-sous-Bois, France

**USNM** Smithsonian Institution, National Museum of National History, Washington, D.C., USA

**ZJPC** Zdeněk Jindra collection, Prague, Czech Republic

## Taxonomy

### Rhyncholepta

Taxon classificationAnimaliaORDOFAMILIA

Bergroth, 1911

Rhyncholepta Bergroth, 1911: 120–121 (description, differential diagnosis). Type species: Rhyncholeptagrandicallosa Bergroth, 1911, by monotypy.
Rhyncholepta : Becker and Grazia-Vieira (1971): 391–393 (redescription, taxonomy, distribution); Rolston (1987): 64 (morphology: lack of parameres); Froeschner (1999): 185 (checklist); Torres Gutiérrez (2005): 70–71, 84, 95: fig. 4.42, 109, 114–115, 118 (diagnosis, key to genera, distribution, record, habitus photo); Greve et al. (2013): 2, 3, 5, 12: fig. 4L, 14, 16: fig. 64 (cladistic analysis, morphology of male genitalia); Cambra et al. (2018): 13, 17: fig. 37 (list, habitus photo); Rider et al. (2018): 66, 81, 110, 190: fig. 2.21 (tribal placement).

#### Redescription.

**Figures 1–4.**
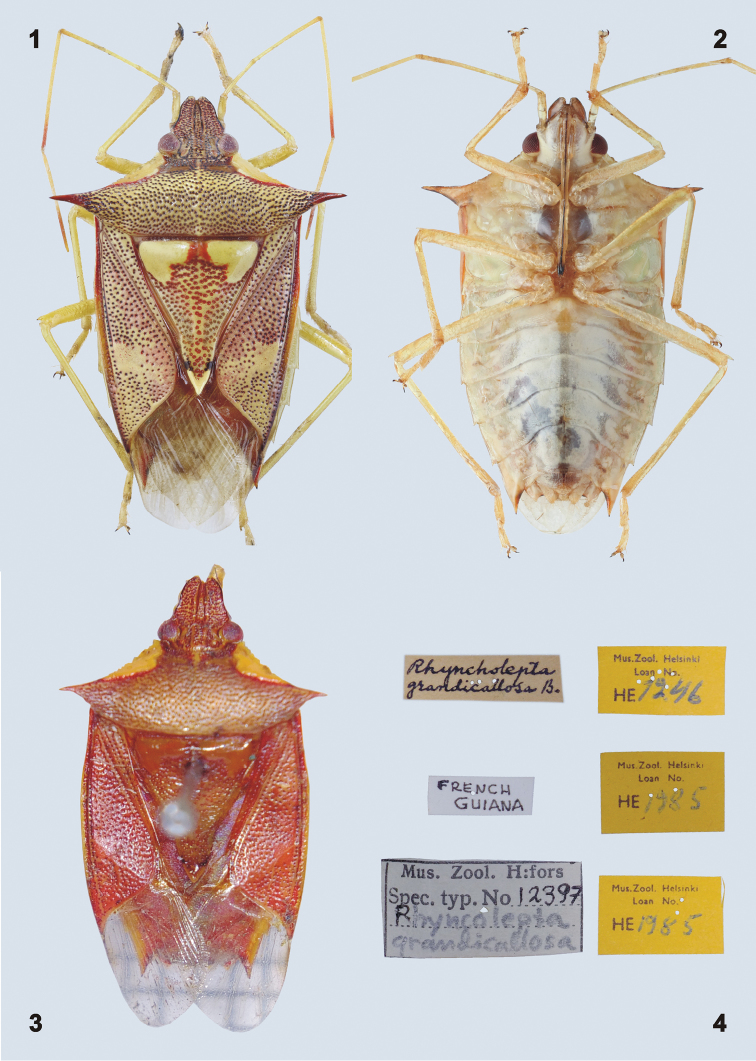
Habitus of *Rhyncholeptagrandicallosagrandicallosa* Bergroth, 1911. **1** candidate neotype, ♂, French Guiana, Camp Caimans **2** ♀, ventral view, French Guiana, Camp Caimans **3** lectotype, ♀, French Guiana **4** labels of the lectotype. (photographs **1, 2** – P. Kment; **3, 4** – A. Albrecht)

**Figures 5–8.**
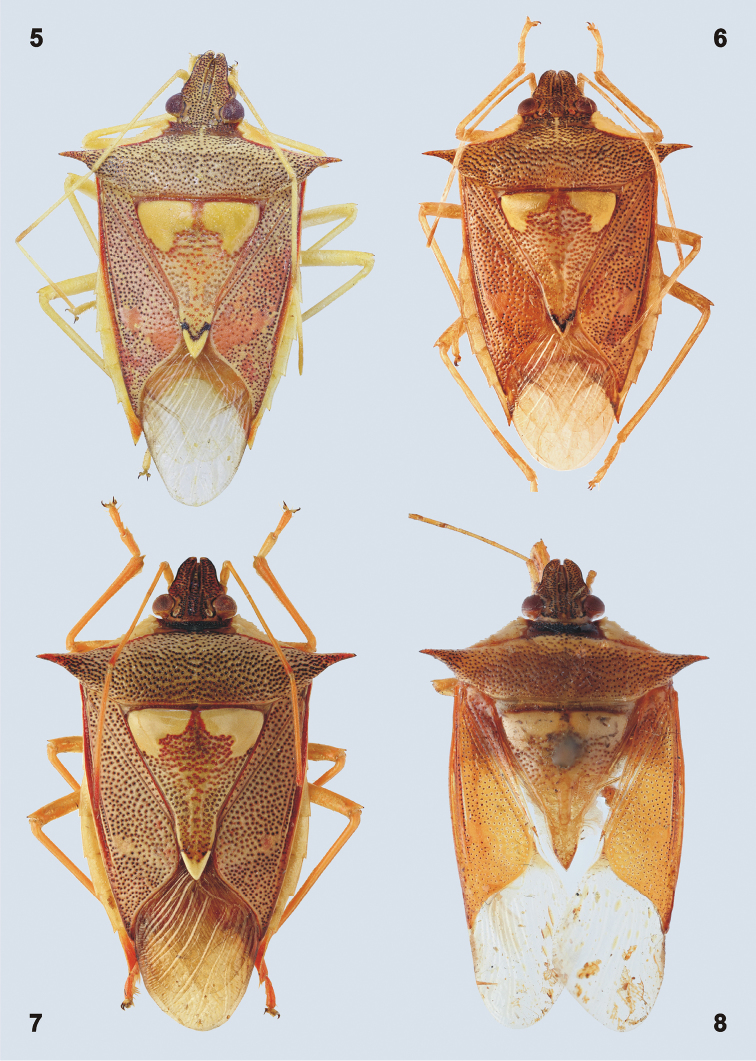
Habitus of *Rhyncholepta* species. **5***Rh.grandicallosacentroamericana* subsp. n., paratype, ♂, Panama, Pipeline Road **6***Rh.henryi* sp. n., holotype, ♂, French Guiana, Camp Caimans **7***Rh.meinanderi* Becker & Grazia-Vieira, 1971, ♂, Ecuador, Arajuno env. **8***Rh.wheeleri* sp. n., holotype, ♂, Guyana. (photographs P. Kment)

***Coloration.*** Dorsal surface of body (Figs 1, 3, 5–8) reddish to reddish brown with large callosities on anterolateral region of pronotum and anterolateral angles of scutellum, small callosity at apex of scutellum, and entire connexiva yellowish (in living specimens phosphorous greenish yellow); impunctate spot on corium appears more or less yellowish red according to specimen. Dorso- and ventrolateral margins of mandibular plates each with a narrow black stripe, head along inner margin of compound eyes with fine black line in some specimens. Scutellum ante-apically with black V-shaped spot which may be reduced to a small black dot on each side of the apex (Figs 14–25). Antennae and legs yellowish (sometimes slightly reddish), apices of basi- and distiflagellum sometimes contrastingly red or brownish. Hypocostal lamina of hemelytron and ventral surface of the body yellowish (Figure 2). Apex of rostrum and tarsal claws distally black. Membrane colorless, hyaline, and translucent. Abdominal terga reddish yellow.

***Structure.*** Body elongate, deltoid, widest across humeral angles and narrowing anteriad and posteriad (Figs 1–2, 5–7). Dorsal surface of body slightly convex, venter strongly convex. Body length 11.22–14.00 mm.

**Figures 9–13.**
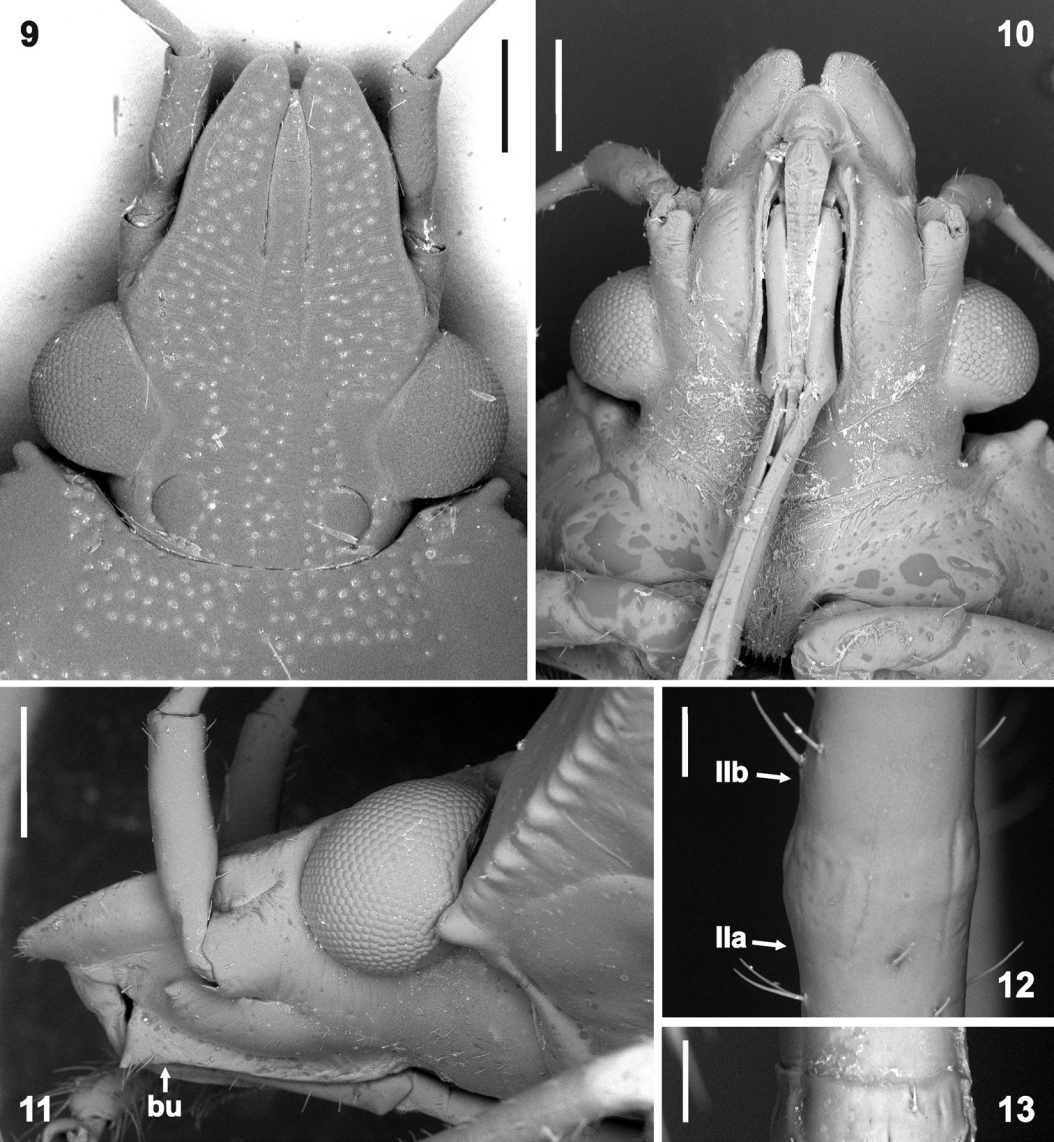
Morphology of *Rhyncholepta* species. **9, 11–12***Rh.grandicallosagrandicallosa* Bergroth, 1911, ♂, French Guiana, Camp Caimans **10, 13***Rh.grandicallosacentroamericana* subsp. n., paratype, ♂, Costa Rica, Rancho Quemado. **9** head, dorsal view (magnification 37×) **10** head, ventral view (37×); **11** head, lateral view (50×) **12–13** joint between basipedicellite and distipedicellite (**12** lateral view, 300×; **13** ventral view, 300×). Abbreviations: **bu** buccula, **IIa** basipedicellite, **IIb** distipedicellite. Scale bars: 0.5 mm (**9–11**); 50 μm (**12–13**). (micrographs P. Kment)

*Head* (Figs 9–11) roughly triangular, approximately as long as wide across eyes (ca. 1.1 : 1.0), compound eyes large, exceeding head outline laterally by about half their width, dorsal surface of head flat. Mandibular plates continually narrowing from eyes toward apices, lateral margins slightly concave at midlength and slightly convex in anterior half, surpassing clypeus by about basal width of clypeus but not meeting each other, leaving narrow, V-shaped notch in front of clypeus (Figure 9; width of notch varies intraspecifically). Clypeus sharply narrowing in anterior half, apically free but slightly depressed compared to mandibular plates. Ocelli large, posteromedial to compound eyes, distance between ocellus and adjacent compound eye about diameter of ocellus, distance between ocelli about three diameters (Figure 9). Antenniferous tubercles (Figs 9–11) short, completely visible in dorsal view, without spine or tubercle laterally. Antennae long, surpassing apex of scutellum when folded backwards (Figure 5), pentamerous, scape (I) short, reaching ca. apex of head, cylindrical, stout (about twice diameter of basipedicellite), remaining antennomeres slender, narrowly cylindrical, basipedicellite (IIa) longer than scape but about half the length of distipedicellite (IIb), basiflagellum (III) and distiflagellum (IV), all nearly same length (for exact lengths see Table 1). Joint between basipedicellite and distipedicellite inconspicuous, more or less fused (Figs 12–13). Bucculae (Figs 10–11) short, low, anteriorly rectangular without spine or produced into a short acutangulate spine (varies intraspecifically), posteriorly reaching about anterior margin of eye, evanescent (Figure 11). Apex of rostral segment I slightly surpassing bucculae (Figs 10–11), apex of rostrum reaching anterior margin of metacoxae (Figure 2); length ratio of rostral segments: II > I = III > IV, II about twice length of segment IV.

**Table 1.** Measurements of *Rhyncholepta* species-group taxa.

Measurement (mm): median, minimum–maximum, n of specimens	* Rhyncholepta grandicallosa grandicallosa *		* Rhyncholepta grandicallosa centroamericana *		* Rhyncholepta henryi *		* Rhyncholepta meinanderi *		* Rhyncholepta wheeleri *
	male	female	male	female	male	female	male	female	male
Total body length	12.79	11.95	12.66	12.67	12.18	12.20	12.55	12.50	13.17
	**11.75–13.30**	**11.25–12.53**	**11.22–13.44**	**11.29–14.00**	**11.44–12.88**	**11.70–12.80**	**12.50–13.90**	**12.20–13.74**	
	n = 9	n = 10	n = 10	n = 10	n = 9	n = 10	n = 5	n = 6	n = 1
Body length to segment VII	11.31	11.19	10.92	11.90	10.78	11.23	11.20	11.25	–
	**10.50–12.30**	**10.25–11.70**	**10.01–11.68**	**10.61–12.80**	**10.00–11.24**	**10.90–11.45**	**10.70–11.90**	**10.70–11.40**	
	n = 10	n = 10	n = 10	n = 10	n = 10	n = 10	n = 7	n = 8	n = 1
Head length	2.41	2.45	2.46	2.55	2.50	2.50	2.48	2.51	2.65
	**2.22–2.50**	**2.30–2.60**	**2.24–2.70**	**2.27–2.76**	**2.34–2.65**	**2.35–2.64**	**2.20–2.55**	**2.35–2.76**	
	n = 10	n = 10	n = 10	n = 10	n = 10	n = 10	n = 7	n = 10	n = 1
Head width	2.31	2.30	2.34	2.40	2.35	2.38	2.30	2.34	2.40
	**2.21–2.37**	**2.25–2.40**	**2.12–2.42**	**2.21–2.53**	**2.27–2.49**	**2.30–2.45**	**2.25–2.45**	**2.25–2.42**	
	n = 10	n = 10	n = 10	n = 10	n = 10	n = 10	n = 7	n = 10	n = 1
Interocular width	1.05	1.05	1.08	1.16	1.12	1.05	1.10	1.10	1.18
	**0.95–1.16**	**1.00–1.15**	**1.04–1.15**	**1.03–1.21**	**1.01–1.19**	**1.00–1.21**	**1.00–1.14**	**1.00–1.17**	
	n = 10	n = 10	n = 10	n = 10	n = 10	n = 10	n = 7	n = 10	n = 1
Pronotum length	2.49	2.45	2.50	2.59	2.47	2.45	2.65	2.53	2.65
	**2.35–2.70**	**2.30–2.65**	**2.28–2.71**	**2.36–2.84**	**2.26–2.65**	**2.10–2.55**	**2.51–2.80**	**2.40–2.70**	
	n = 10	n = 10	n = 10	n = 10	n = 10	n = 10	n = 7	n = 10	n = 1
Pronotum width	8.36	7.99	7.69	8.00	7.67	7.88	8.80	8.43	8.14
	**7.93–8.70**	**7.75–8.75**	**6.85–8.24**	**7.03–8.68**	**7.53–8.10**	**7.55–8.10**	**8.40–9.85**	**7.85–8.85**	
	n = 10	n = 10	n = 10	n = 10	n = 10	n = 10	n = 7	n = 10	n = 1
Scutellum length	4.44	4.26	4.41	4.50	4.26	4.28	4.70	4.45	4.61
	**4.25–4.75**	**4.10–4.42**	**3.97–4.69**	**4.08–4.85**	**4.04–4.45**	**4.05–4.34**	**4.55–5.01**	**4.30–4.70**	
	n = 10	n = 10	n = 10	n = 10	n = 10	n = 10	n = 7	n = 10	n = 1
Scutellum width	3.77	3.60	3.73	3.83	3.53	3.70	3.90	3.80	3.73
	**3.53–3.91**	**3.40–3.93**	**3.39–3.90**	**3.44–4.10**	**3.10–3.69**	**3.60–3.76**	**3.80–4.15**	**3.50–4.00**	
	n = 10	n = 10	n = 10	n = 10	n = 10	n = 10	n = 7	n = 10	n = 1
Scape (I) length	0.79	0.75	0.76	0.77	0.79	0.80	0.79	0.78	0.78
	**0.69–0.87**	**0.67–0.80**	**0.56–0.84**	**0.64–0.84**	**0.66–0.86**	**0.63–0.85**	**0.70–0.90**	**0.66–0.89**	
	n = 10	n = 10	n = 10	n = 10	n = 10	n = 10	n = 7	n = 10	n = 1
Basipedicellite (IIa) length	1.25	1.22	1.32	1.33	1.30	1.20	1.30	1.23	1.22
	**1.12–1.38**	**1.14–1.36**	**1.16–1.43**	**1.21–1.44**	**1.08–1.48**	**1.10–1.25**	**1.18–1.50**	**1.15–1.46**	
	n = 10	n = 10	n = 10	n = 10	n = 10	n = 10	n = 7	n = 10	n = 1
Distipedicellite (IIb) length	2.55	2.35	2.50	2.44	2.56	2.55	2.70	2.52	2.63
	**2.38–2.70**	**2.10–2.40**	**2.05–2.73**	**1.95–2.78**	**2.40–2.80**	**2.30–2.70**	**2.60–3.00**	**2.30–2.82**	
	n = 10	n = 10	n = 10	n = 10	n = 10	n = 10	n = 7	n = 10	n = 1
Basiflagellum (III) length	3.37	3.18	3.06	3.12	3.50	3.50	3.65	3.44	–
	**2.96–3.60**	**3.05–3.35**	**2.60–3.44**	**2.68–3.43**	**3.20–3.65**	**3.12–3.70**	**3.40–3.95**	**3.25–3.60**	
	n = 10	n = 9	n = 10	n = 8	n = 10	n = 10	n = 7	n = 8	
Distiflagellum (IV) length	2.93	2.65	2.58	2.64	2.76	2.95	3.13	3.00	–
	**2.72–2.95**	**2.60–2.71**	**1.89–2.78**	**2.33–2.77**	**2.67–2.95**	**2.72–3.15**	**2.90–3.20**	**2.80–3.15**	
	n = 7	n = 5	n = 8	n = 7	n = 5	n = 9	n = 6	n = 7	

*Thorax.* Pronotum (Figs 1, 3, 5–8) trapezoid with prominent humeral angles, each bearing a stout, sharp spine directed laterad. Anterior pronotal margin concave to receive postocular portion of head (Figure 9); each anterolateral angle nearly rectangular, apically with small rounded tubercle (Figs 9, 11); anterolateral margins straight, slightly crenulate (Figure 11), without emargination or carina; posterolateral margins shallowly sinuate; posterolateral angles obtusangulate; posterior margin straight. Pronotal surface simply convex, anterior to humeral angles, sloping toward head.

Scutellum (Figs 1, 3, 5–8) longer than wide, triangular, apically acutangulate, surface convex in frenal portion (appearing slightly gibbose near anterolateral callosities), postfrenal portion flat, apex with more or less prominent V-shaped callosity (Figs 14–25).

**Figures 14–25.**
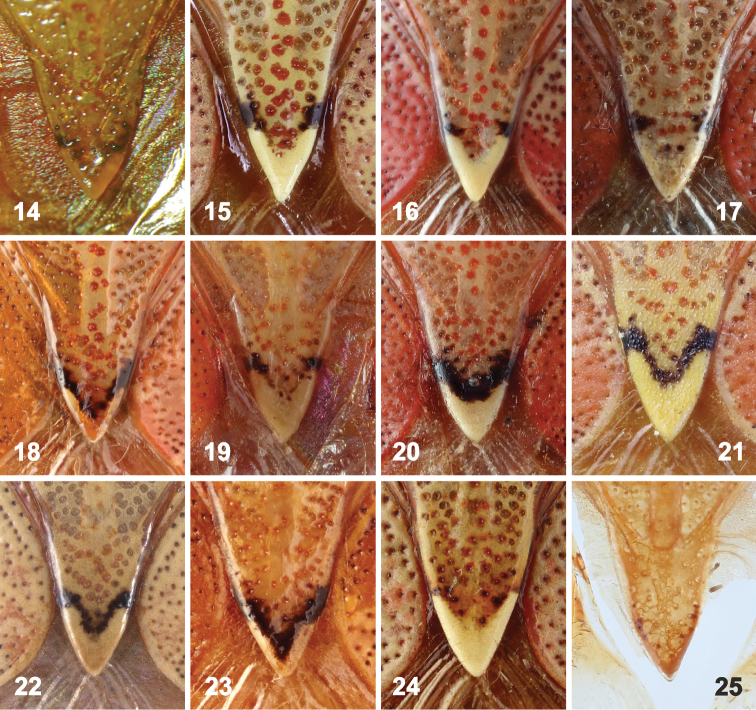
Apex of scutellum of *Rhyncholepta* species. **14–18***Rh.grandicallosagrandicallosa* Bergroth, 1911: 14 – ♀, lectotype, French Guiana **15** ♂, candidate neotype, French Guiana, Camp Caimans **16** ♂, French Guiana, Camp Voltaire **17–18** French Guiana, Camp Caimans (**17** ♀, **18** ♂). **19–22***Rh.grandicallosacentroamericana* subsp. n.: **19** ♀, Panama, Barra del Colorado **20** ♀, Costa Rica, Rancho Quemado **21** ♂, Panama, Pipeline Road **22** Guatemala, Firmeza. **23***Rh.henryi* sp. n., holotype, ♂, French Guiana, Camp Caimans. **24***Rh.meinanderi* Becker & Grazia-Vieira, 1971, ♂, Ecuador, Arajuno env. **25***Rh.wheeleri* sp. n., holotype, ♂, Guyana. (photographs **14** A. Albrecht; **15– 21, 23–25** P. Kment; **22** J.E. Eger)

Clavus narrow, anteriorly with maximally 5 rows of punctures, narrowing towards frena (Figs 1, 5, 7). Lateral margins of corium narrowing posteriad. Anterodistal angle of each corium (appearing posterolateral in resting position!) sharply acutangulate, far surpassing apex of scutellum, reaching middle of connexival segment VII (Figs 5–6); posterodistal angle rounded, distal (membranal) margin concave (Figs 5, 7). Membrane surpassing apex of abdomen (about one third of its length), with numerous longitudinal, parallel veins branching from basal transverse vein (Figure 7).

**Figures 26–29.**
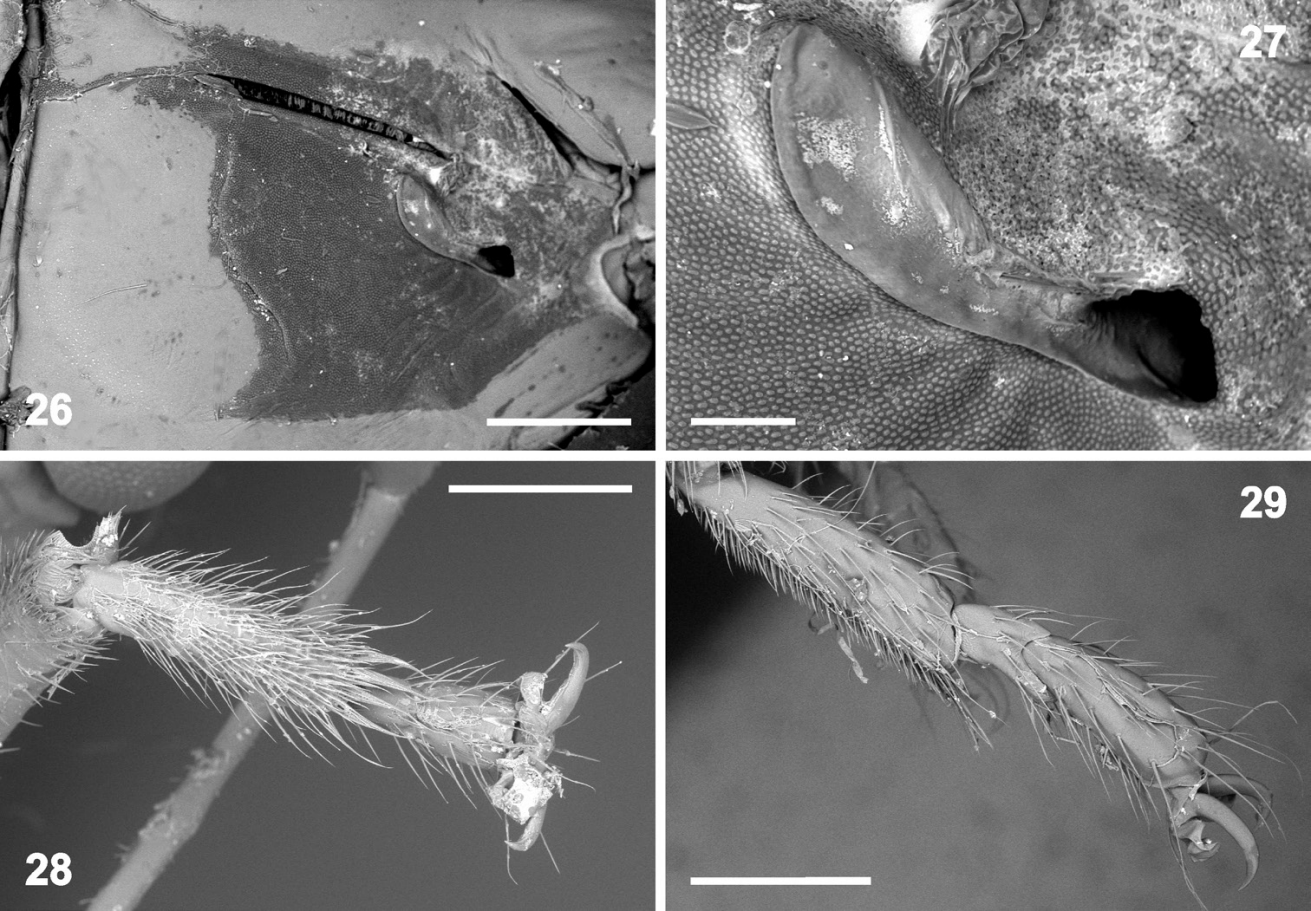
Morphology of *Rhyncholepta* species. **26–28***Rh.grandicallosacentroamericana* subsp. n.: ♂, Costa Rica, Rancho Quemado: **26** external scent efferent system of metathoracic scent gland (magnification 55×) **27** peritreme and ostiole (200×) **28** protarsus, ventral view (70×) **29***Rh.grandicallosagrandicallosa* Bergroth, 1911, ♂, French Guiana, Camp Caimans, metatarsus, lateral view (70×). Scale bars: 0.5 mm (**26, 28–29**); 100 μm (**27**). (micrographs: P. Kment)

Mesosternum with low median carina, most prominent anteriorly; metasternum hexagonal, flat. Each ostiole between meso- and metacetabulum, small, oval (Figure 26), opening posterolaterad (invisible in ventral view); periostiolar depression small (Figure 27). Peritreme in form of short groove, slightly curved anterolaterad, apically rounded and elevated above surrounding pleuron (Figs 26, 27). Metapleural evaporatoria large, each occupying nearly inner two thirds of metapleuron, laterally emarginated by low sinuate carina (Figure 26); mesopleural evaporatoria small, each limited to posterior margin (narrowly reaching posterolateral angle of mesopleuron) and not well delimited (Figure 26).

Femora slender, cylindrical (Figure 2), with short, stout spine dorsoapically (Figs 1, 2, 5–7), ventral surface unarmed. Tibiae slender, rounded, only slightly flattened dorsoapically, without spines or impressed lines on outer surfaces. Length of tarsal segments I > III > II, I about as long as II and III combined (Figure 29).

*Abdomen* with ventral surface regularly convex, without median keel or groove (Figure 2). Abdominal segment III anteromedially with low, broadly rounded protuberance not reaching between metacoxae (Figure 2). Abdominal segments III–VII with anterior margins convex and posterior margins concave medially, more pronounced posteriad; segment VII anteriorly distinctly produced forwards, longer medially than preceding segments (Figure 2). Spiracles concolorous with surrounding abdominal surface, each not surrounded by a callosity. Trichobothria 2 + 2 on each abdominal segment, arranged immediately behind stigma, one on each side.

Lateral margins of connexivum exposed in dorsal view (Figs 1, 5–7). Posterolateral angles of connexival segment III rectangular, not produced, posterolateral angles of following segments more prominent, becoming acutangulate on segment VI; posterolateral angles of segment VII acutangulate, distinctly produced posteriad and surpassing posterior margins of genital segments in male (Figure 1) and female (Figs 2, 90–93).

*Male genitalia.* Genital capsule (Figs 30, 32, 34, 36, 38, 40) relatively large, as long as or slightly shorter than wide; width of genital capsule 2.2–2.5 mm. Dorsal wall (Figs 54, 56, 58, 60, 62, 64, 66–71) rather short, gibbose, simple. Ventral wall (e.g. Figs 30, 32, 34, 36, 38, 40, 66–71) produced posteriad with large depression anteapically. Posterolateral angles of genital capsule each with thin wall, opened dorsally and rounded posteriorly (e.g. Figs 54, 56, 60), space between posterolateral angles filled by ventral rim. Ventral rim expanded dorsally and anteriorly forming complicated hypandrium (of species-specific shape) (e.g. Figs 42, 48, 54, 60, 66), wider than long, symmetrical along median axis and bearing three short-to-long, narrow-to-large projections (posterior, lateral, and anterior) on each side (e.g. Figs 55, 57, 59, 61, 63, 65, 72–77). Proctiger simple. Parameres lacking. Phallus (Figs 78–89) (description follows Becker and Grazia-Vieira 1971: figs 6–11; with original terminology in parentheses): strongly sclerotized basal plates of articulatory apparatus joined by ponticulus basilaris ventrally, with degree of sclerotization equal to that of basal plates; dorsal connectives almost as wide as lateral areas of basal plates; capitate process (= processus capitatus) well developed; phallotheca cylindrical, with pair of small dorsal processes at base; endophallic (= ejaculatory) reservoir voluminous; aedeagus (= vesica) more or less sinuous, contained within conjunctiva, with apical opening through which phallotreme (= secondary gonopore) emerges (Figs 79, 82, 85, 88).

**Figures 30–35.**
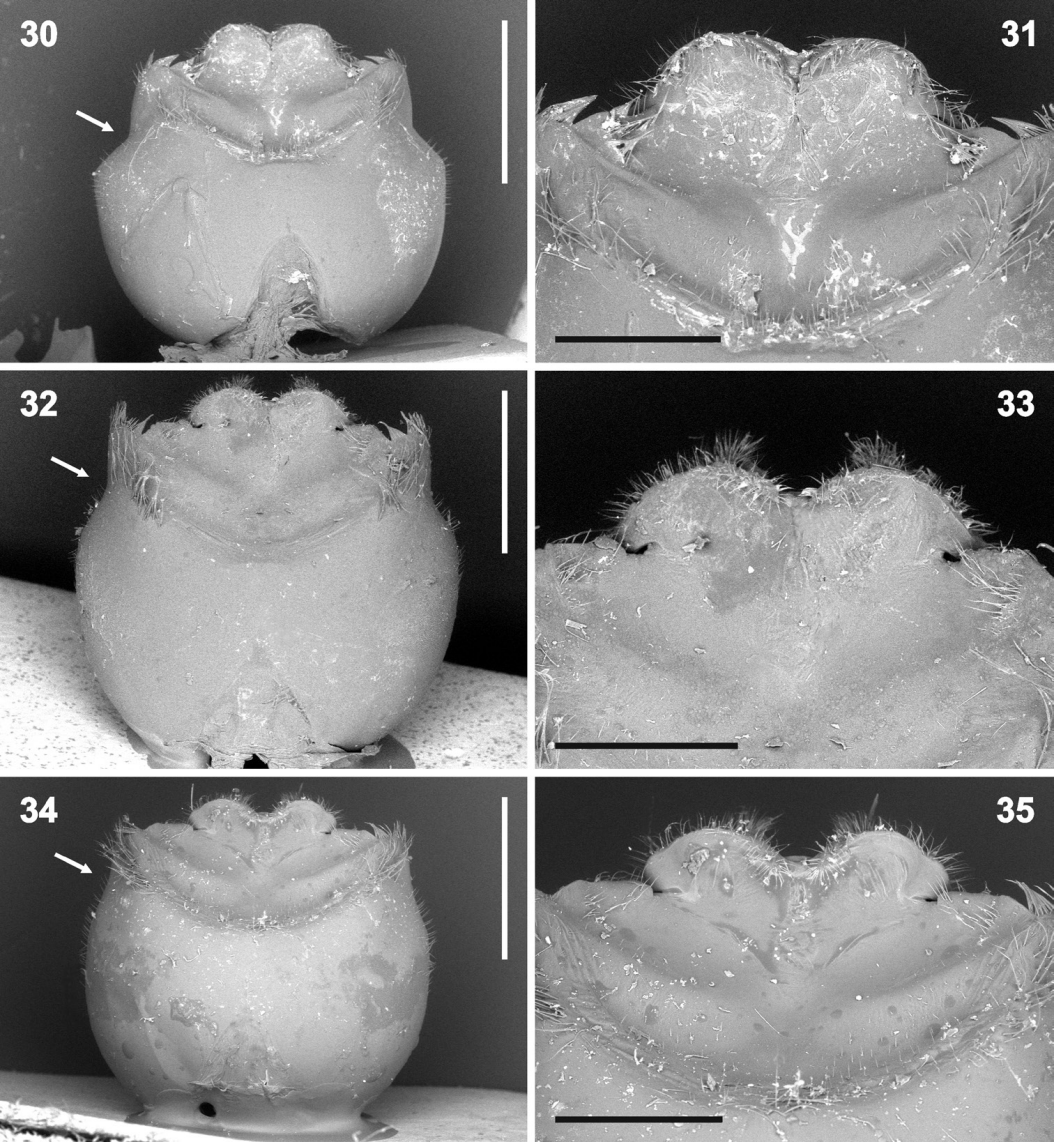
Genital capsule (**30, 32, 34** magnification 32×) and detail of hypandrium (**31, 33, 35** 80×) in ventral view. **30–31***Rh.grandicallosagrandicallosa* Bergroth, 1911, French Guiana, Camp Caimans **32–35***Rh.grandicallosacentroamericana* subsp. n.: **32–33** Panama, Pipeline Road **34–35** Costa Rica, Rancho Quemado. Scale bars: 1 mm (**30, 32, 34**); 0.5 mm (**31, 33, 35**). (micrographs: P. Kment)

**Figures 36–41.**
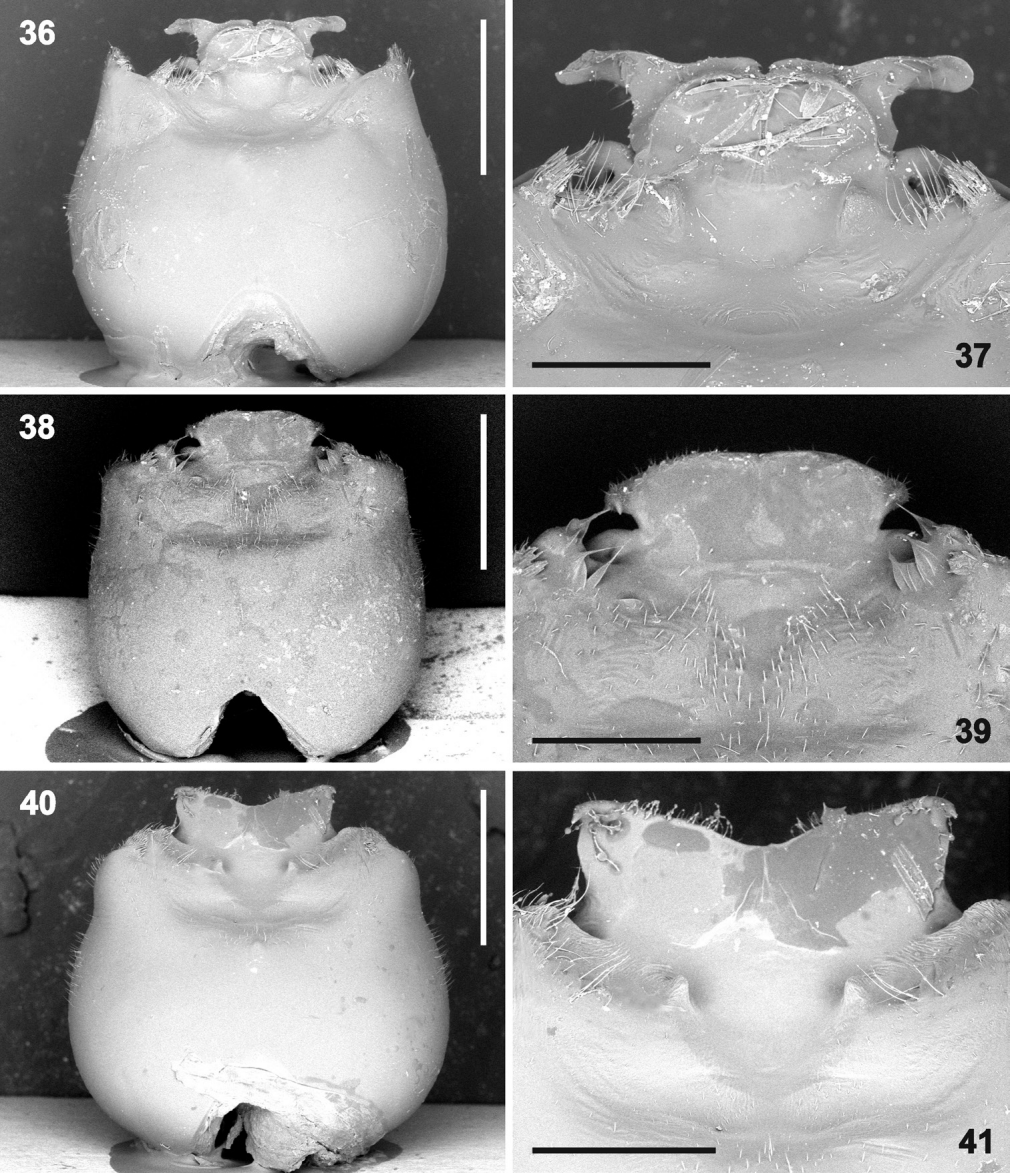
Genital capsule (**36, 38, 40** magnification 32×) and detail of hypandrium (**37, 39, 41** 80×) in ventral view. **36–37***Rh.henryi* sp. n., holotype, French Guiana, Camp Caimans **38–39***Rh.meinanderi* Becker & Grazia-Vieira, 1971, Ecuador, Yasui NP **40–41***Rh.wheeleri* sp. n., holotype, Guyana. Scale bars: 1 mm (**36, 38, 40**); 0.5 mm (**37, 39, 41**). (micrographs P. Kment)

*Female genitalia* (description follows Becker and Grazia-Vieira 1971: figs 12–17; original terminology in parentheses): External female genitalia (Figs 90–93) with posterior margins of laterotergites VIII and IX acutangulate; posterior margins of valvifers (= gonocoxae) VIII straight; valvulae (= gonapophyses) VIII fused medially, forming triangulum; valvifers (= gonocoxites) IX fused along median line forming partially covered plate-like sclerite (= pseudosternite); valvulae IX fused medially, forming single piece as wide as valvifers IX. Internal female genitalia (Becker and Grazia-Vieira 1971: figs 14–17): Dorsal wall of gynatrium (= pars communis) showing a thickening of vaginal intima around spermathecal opening (= orifice receptaculi). Spermatheca (= receptaculum seminis): Proximal duct (= ductus receptaculi anterior) much longer and thinner than distal duct (= ductus receptaculi posterior), distal duct widening towards proximal flange; spermathecal dilation well developed; intermediate part of spermatheca with proximal flange narrower than distal flange, apical receptacle (= capsula seminalis) subglobular with three hook-shaped projections.

***Vestiture.*** Body appearing bare, but pro-, meso- and metapleura with very short adpressed pale setae (invisible in greasy specimens). Antennae and legs with short semi-erect pale setae, at least slightly shorter than diameter of particular antennomere or tibia. Abdomen ventrally along midline and external female genitalia with sparse long, erect, pale setae. Genital capsule with short, erect, pale hairs around anteapical depression on ventral wall (Figs 32, 39, 44, 67), along margins of genital aperture (Figs 55, 63), on posterloateral angles (Figs 32, 52) and on hypandrium (Figs 43, 55, 73, 77). Apical surface of head (except small area medially of compound eyes) (Figure 9), pronotum (except large anterolateral callosities, cicatrices and humeral spines), clavus, and corium (except round spot on corial disc at level of postfrenal portion of scutellum) (Figs 1, 5, 7) regularly covered with black punctures, only punctures near posterolateral angle of each corium distinctly smaller and sometimes concolorous. Disc of scutellum (except pair of anterolateral and one apical callosity) with large, reddish to concolorous punctures (those near V-shaped line sometimes black). Connexivum and legs with very small concolorous punctures. Callosities of pronotum and scutellum (Figure 1) smooth, lustrous. Pro-, meso- and metapleura with sparse concolorous punctures, best visible laterally on propleuron (punctures near humeral angles sometimes black) and laterally and posteriorly on metapleuron. Ventral surface of head, sterna, abdomen ventrally, genital capsule, and female external genitalia smooth.

**Figures 42–47.**
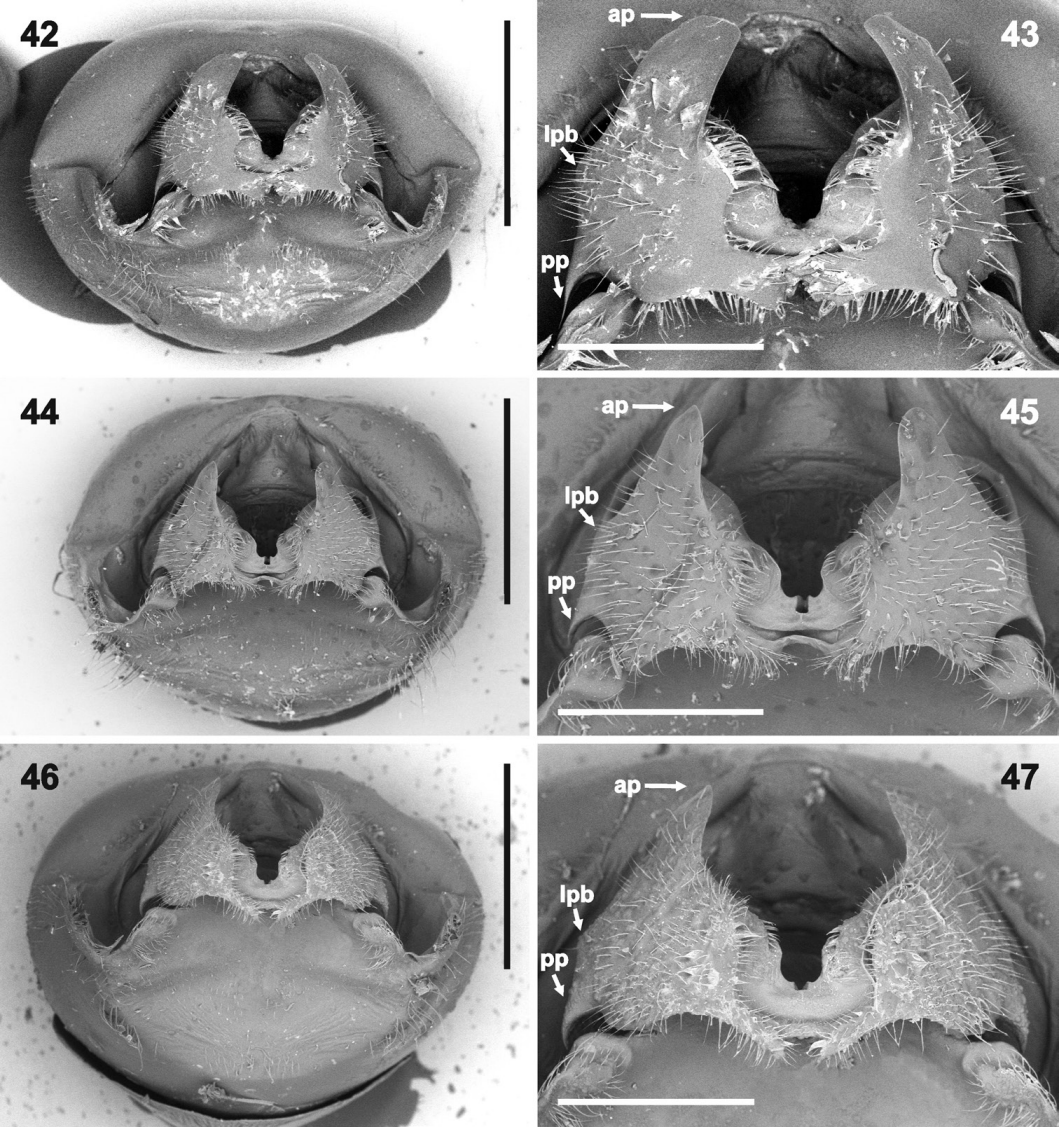
Genital capsule (**42, 44, 46** magnification 45×) and detail of hypandrium (**43, 45** 80×, **47** 90×) in posterior (caudal) view. **42–43***Rh.grandicallosagrandicallosa* Bergroth, 1911, French Guiana, Camp Caimans **44–47***Rh.grandicallosacentroamericana* subsp. n.: **44–45** Panama, Pipeline Road; **46–47** Costa Rica, Rancho Quemado. Abbreviations: **ap** anterior hypandrial projection, **lpb** base of lateral hypandrial projection, **pp** posterior hypandrial projection. Scale bars: 1 mm (**42, 44, 46**); 0.5 mm (**43, 45, 47**). (micrographs: P. Kment)

**Figures 48–53.**
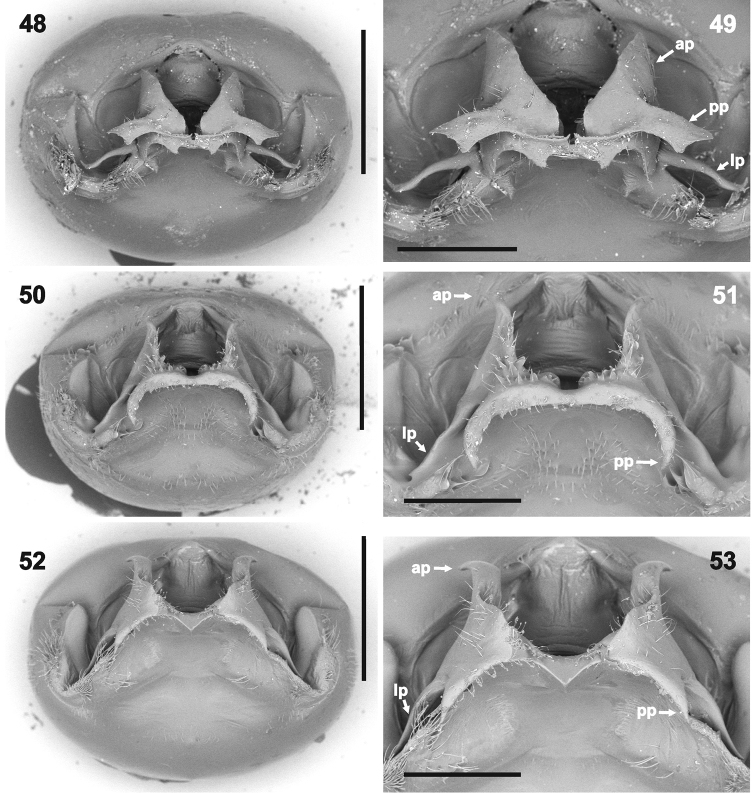
Genital capsule (**48, 50, 52** magnification 45×) and detail of hypandrium (**49, 51, 53** 80×) in posterior (caudal) view. **48–49***Rh.henryi* sp. n., holotype, French Guiana, Camp Caimans **50–51***Rh.meinanderi* Becker & Grazia-Vieira, 1971, Ecuador, Yasui NP **52–53***Rh.wheeleri* sp. n., holotype, Guyana. Abbreviations: **ap** anterior hypandrial projection, **lp** lateral hypandrial projection, **pp** posterior hypandrial projection. Scale bars: 1 mm (**48, 50, 52**); 0.5 mm (**49, 51, 53**). (micrographs: P. Kment)

**Figures 54–59.**
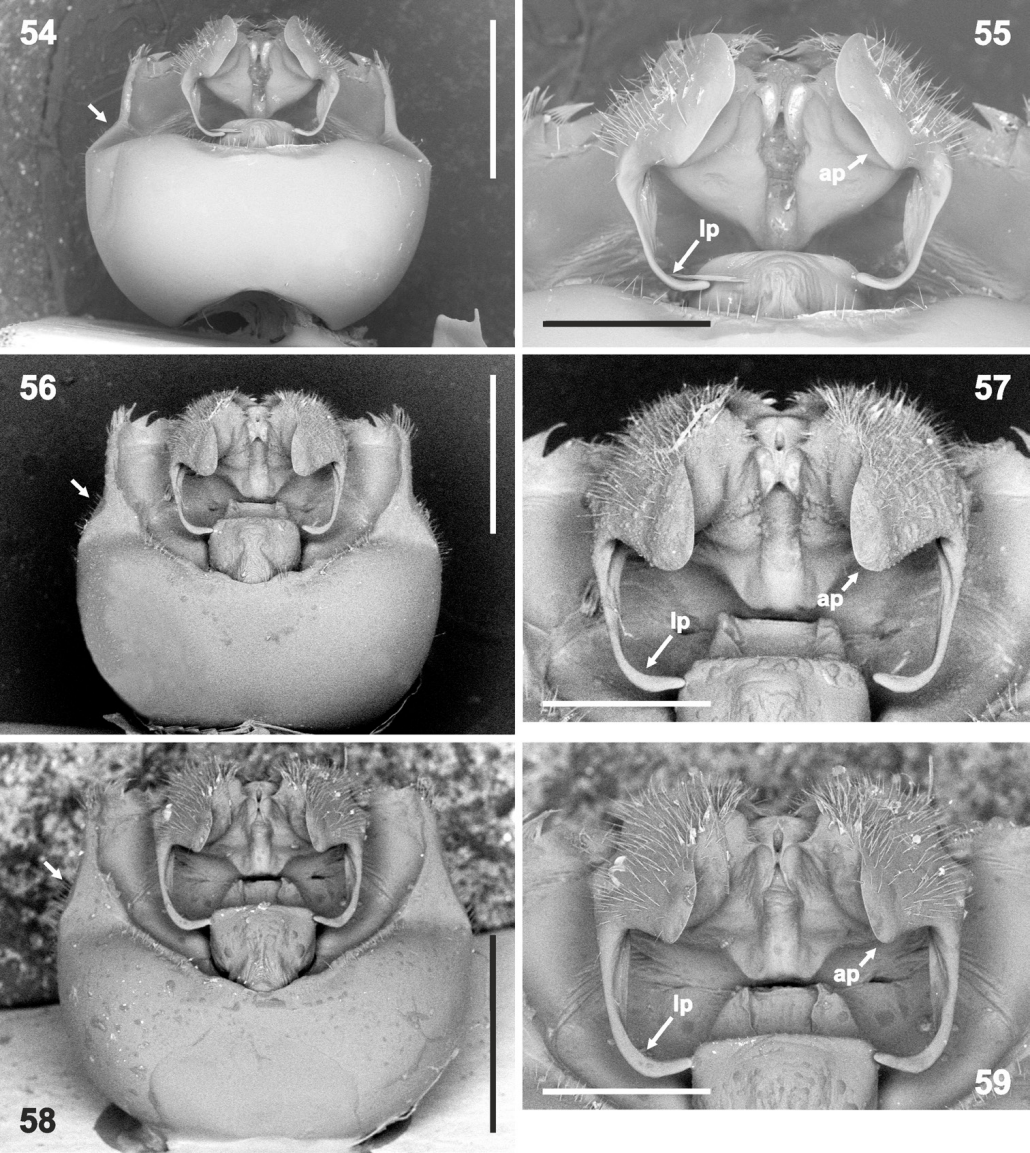
Genital capsule (**54, 56, 58** magnification 32×) and detail of hypandrium (**55, 57, 59** 80×) in dorsal view. **54–55***Rh.grandicallosagrandicallosa* Bergroth, 1911, French Guiana, Camp Caimans **56–59***Rh.grandicallosacentroamericana* subsp. n.: **56–57** Panama, Pipeline Road **58–59** Costa Rica, Rancho Quemado. Abbreviations: **ap** anterior hypandrial projection, **lp** lateral hypandrial projection. Scale bars: 1 mm (**54, 56, 58**); 0.5 mm (**55, 57, 59**). (micrographs: P. Kment)

**Figures 60–65.**
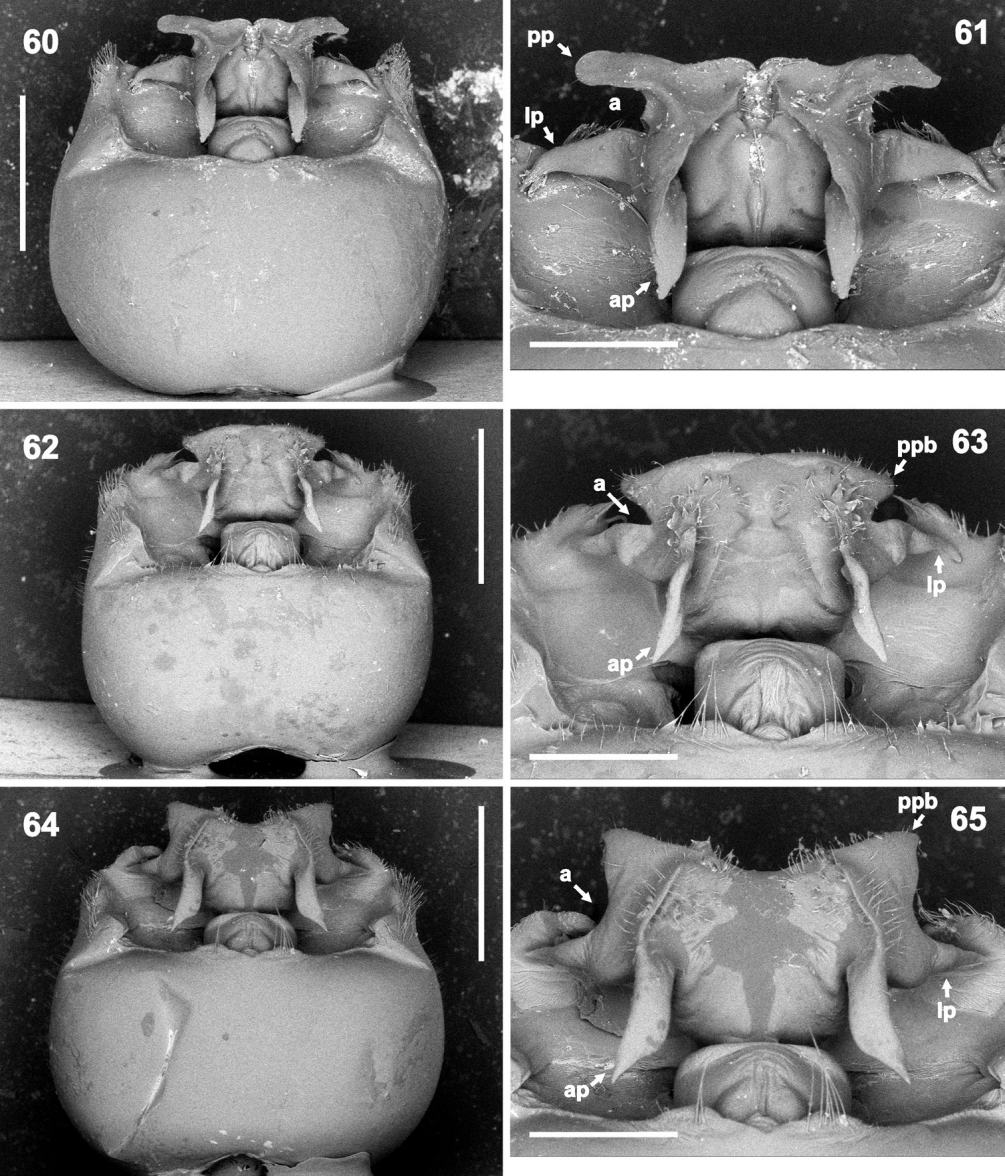
Genital capsule (**69, 62, 64** magnification 32×) and detail of hypandrium (**61, 63, 65** 70×) in dorsal view. **60–61***Rh.henryi* sp. n., holotype, French Guiana, Camp Caimans **62–63***Rh.meinanderi* Becker & Grazia-Vieira, 1971, Ecuador, Yasui NP **64–65***Rh.wheeleri* sp. n., holotype, Guyana. Abbreviations: **a** angle between posterior and lateral hypandrial projection, **ap** anterior hypandrial projection, **lp** lateral hypandrial projection, **ppb** base of posterior hypandrial projection. Scale bars: 1 mm (**60, 62, 64**); 0.5 mm (**61, 63, 65**). (micrographs: P. Kment)

**Figures 66–71.**
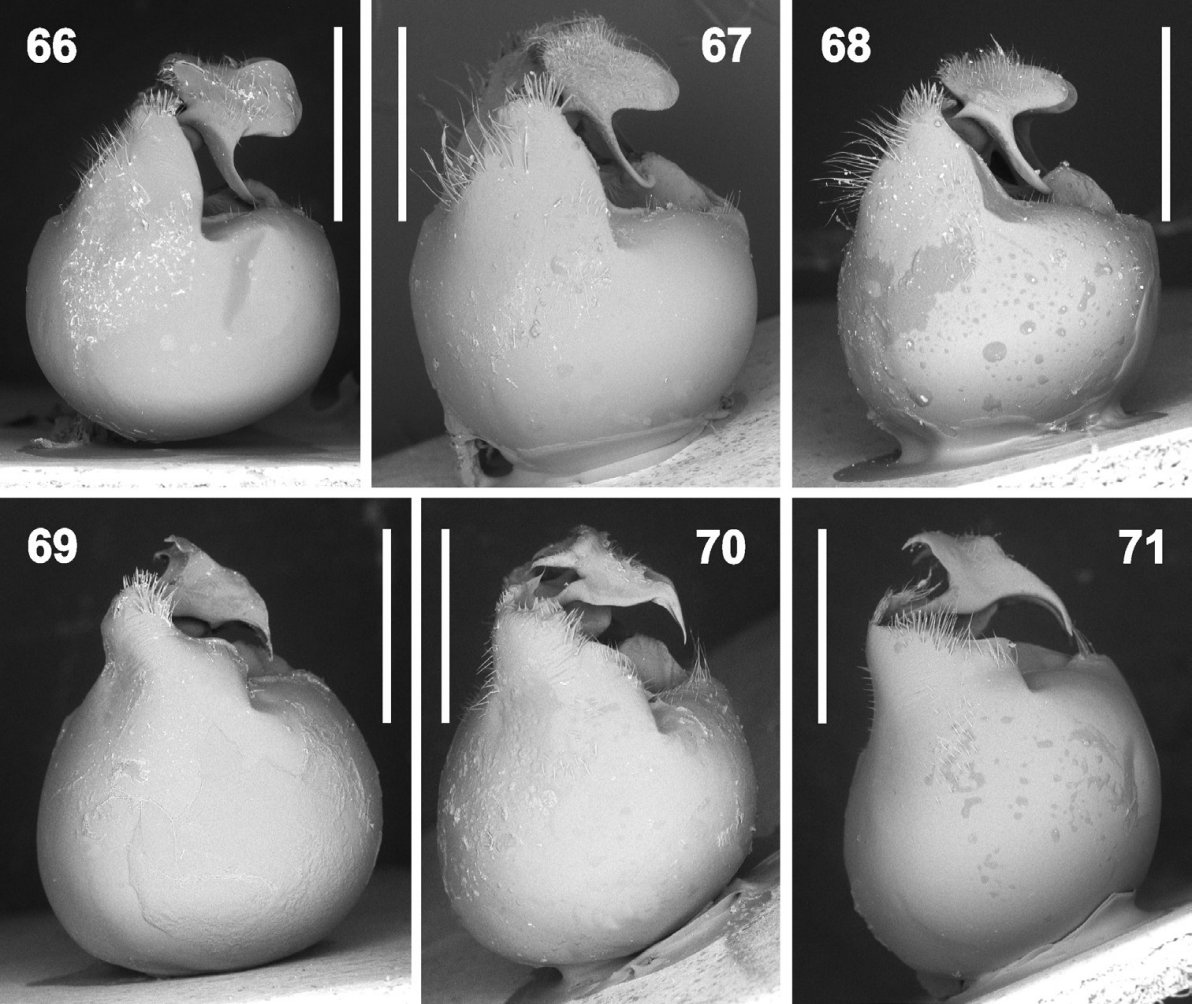
Genital capsule and hypandrium in lateral view (magnification 35×). **66***Rh.grandicallosagrandicallosa* Bergroth, 1911, French Guiana, Camp Caimans **67–68***Rh.grandicallosacentroamericana* subsp. n.: **67** Panama, Pipeline Road **68** Costa Rica, Rancho Quemado **69***Rh.henryi* sp. n., holotype, French Guiana, Camp Caimans **70***Rh.meinanderi* Becker & Grazia-Vieira, 1971, Ecuador, Yasui NP **71***Rh.wheeleri* sp. n., holotype, Guyana. Scale bars: 1 mm. (micrographs P. Kment)

**Figures 72–77.**
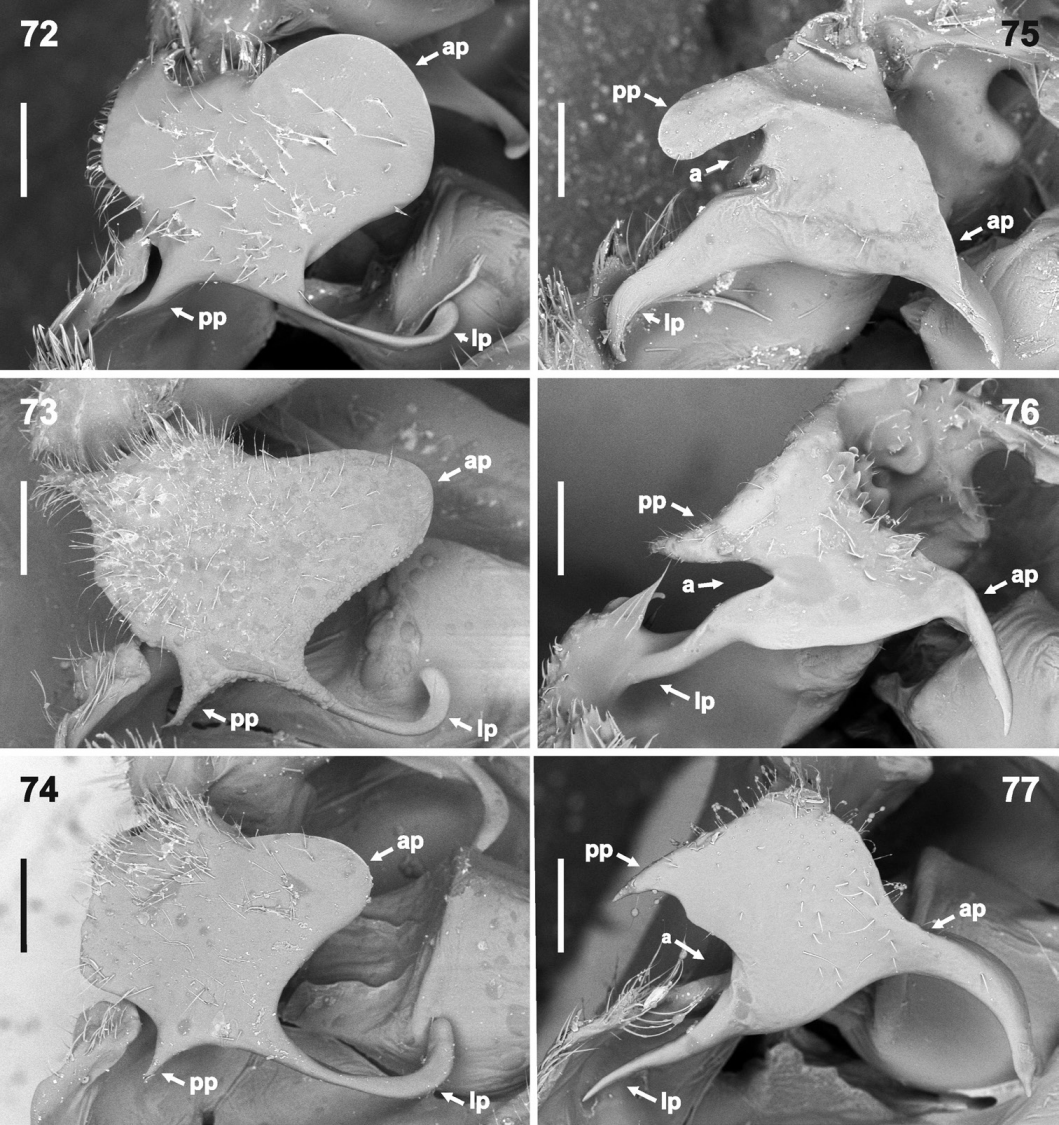
Hypandrial processes in dorso-posterolateral (most exposed) view (magnification 110×). **72***Rh.grandicallosagrandicallosa* Bergroth, 1911, French Guiana, Camp Caimans **73–74***Rh.grandicallosacentroamericana* subsp. n.: **73** Panama, Pipeline Road **74** Costa Rica, Rancho Quemado **75***Rh.henryi* sp. n., holotype, French Guiana, Camp Caimans **76***Rh.meinanderi* Becker & Grazia-Vieira, 1971, Ecuador, Yasui NP **77***Rh.wheeleri* sp. n., holotype, Guyana. Abbreviations: **a** angle between posterior and lateral hypandrial projection, **ap** anterior hypandrial projection, **lp** lateral hypandrial projection, **pp** posterior hypandrial projection. Scale bars: 0.2 mm. (micrographs: P. Kment)

**Figures 78–83.**
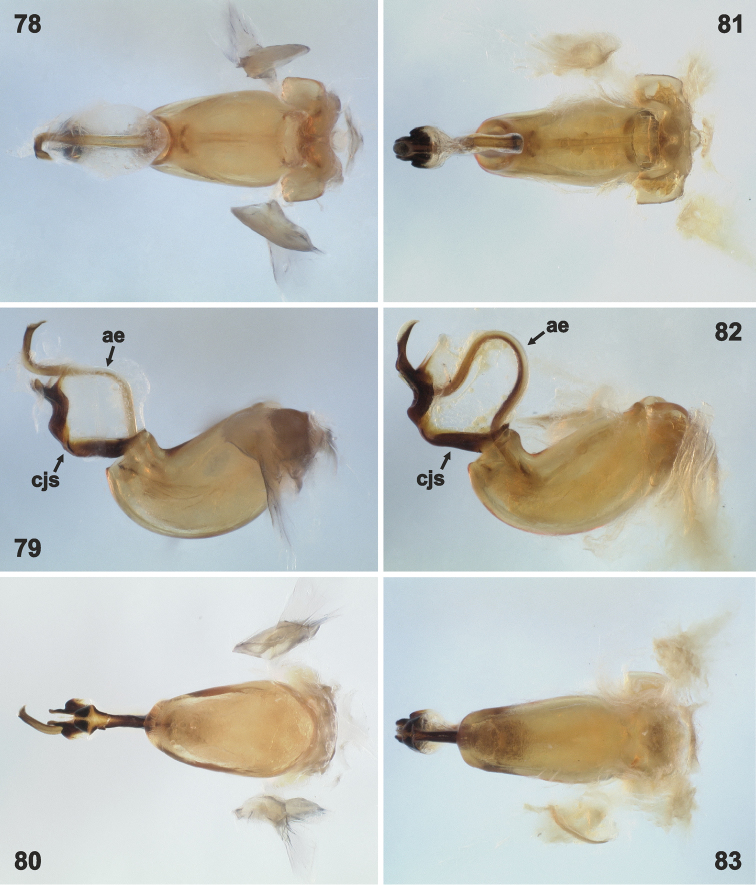
Phallus in dorsal (**78, 81**), lateral (**79, 82**) and ventral (**80, 83**) view. **78–80***Rh.grandicallosagrandicallosa* Bergroth, 1911, French Guiana, Amazone Nature Lodge; **81–83***Rh.grandicallosacentroamericana* subsp. n., Panama, Punta Eseoses. Not to scale. Abbreviations: **ae** aedeagus (= vesica), **cjs** conjunctiva sclerites. (photographs: J.E. Eger)

**Figures 84–89.**
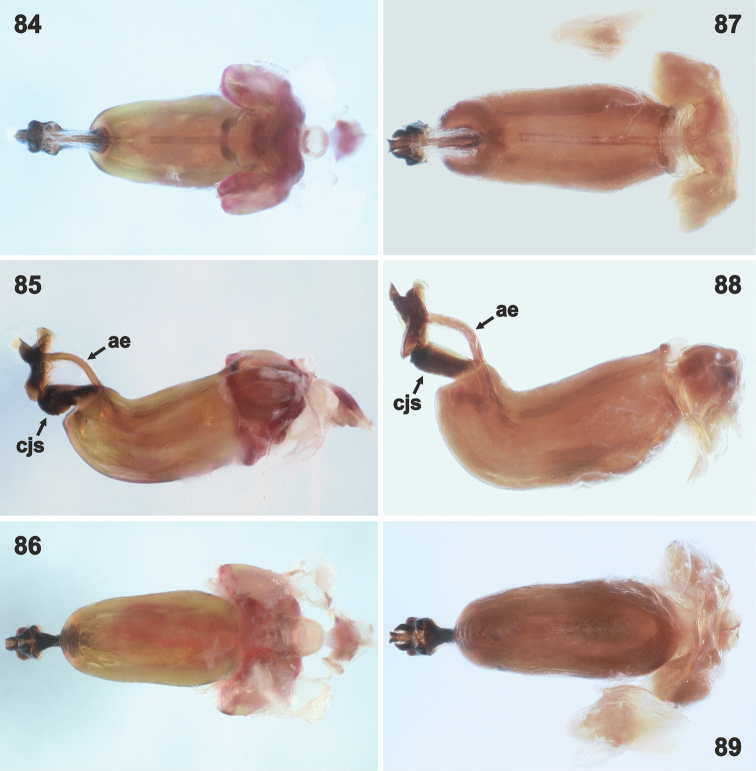
Phallus in dorsal (**84, 87**), lateral (**85, 88**) and ventral (**86, 89**) view. **84–86***Rh.henryi* sp. n., French Guiana, 33 km SE Roura on Kaw Rd. **87–89***Rh.meinanderi* Becker & Grazia-Vieira, 1971, Brazil, near Fzda. Rancho Grande. Not to scale. Abbreviations: **ae** aedeagus (= vesica), **cjs** conjunctiva sclerites. (photographs: J.E. Eger)

**Figures 90–93.**
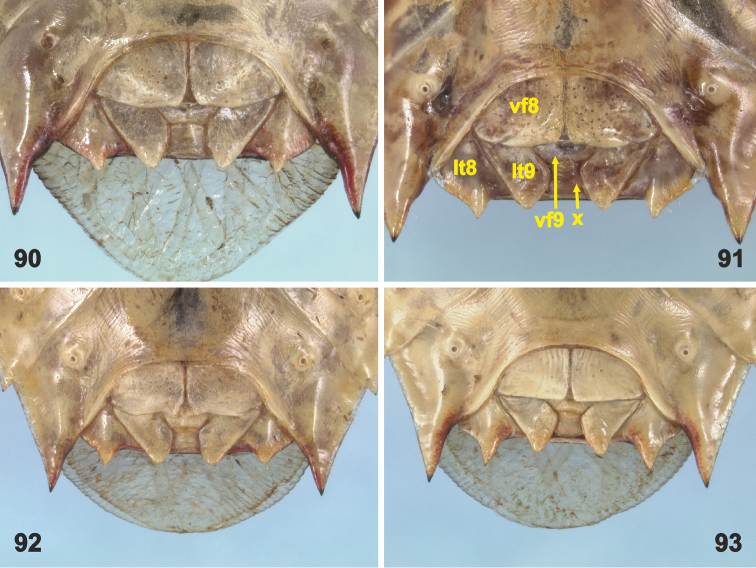
Female external genitalia in ventro-posterior view. **90***Rh.grandicallosagrandicallosa* Bergroth, 1911 **91***Rh.grandicallosacentroamericana* subsp. n. **92***Rh.henryi* sp. n. **93***Rh.meinanderi* Becker & Grazia-Vieira, 1971. Not to scale. Abbreviations: **lt8–9** laterotergites VIII and IX, **vf8–9** valvifers VIII and IX, **x** segment X. (photographs: J.E. Eger)

***Measurements.*** See Table 1. The five species-group taxa we recognize more or less overlap in all measurements that were taken.

#### Differential diagnosis.

Greve (2010) and Rider et al. (2018) characterized members of the tribe Chlorocorini as being medium to large in size, green (fading to yellow after death), and somewhat depressed. They also noted that the head is usually flat dorsally, subtriangular with the apices of the mandibular plates often acute or spinosely produced. The antennae are pentamerous. The anterolateral margins of the pronotum typically are each provided with a row of small to large denticles, and the humeral angles are often prominently spined. The metathoracic scent gland peritremes are usually spout-shaped, relatively short, and do not extend beyond the middle of the metapleuron; the associated evaporatoria typically are large and extensive. The mesosternum is provided with a medial longitudinal carina that does not project forward onto the prosternum. In most included genera, the apex of each femur is provided with a short, dorsal tooth; the tarsi are three-segmented. In all but one included genus, the base of the abdomen is unarmed; that is, lacks a forward-projecting spine or tubercle. Greve (2010) also noted that in most genera, the ventral rim of the pygophore is produced into a process, the so-called hypandrium.

The genus *Rhyncholepta* fits the above criteria except that the head, although relatively flat dorsally and subtriangular, does not have the apices of the mandibular plates acute or spinose, but are narrowly rounded (Figure 9); they typically are reddish brown with pale yellowish green areas in life; the pale areas fade after death.

Rider et al. (2018) included the following genera in Chlorocorini: *Arvelius*, *Chlorocoris*, *Chloropepla*, *Eludocoris*, *Fecelia*, *Loxa*, *Mayrinia*, and *Rhyncholepta*. *Rhyncholepta* can be separated from the above genera by the dorsal coloration (e.g., Figs 1, 3, 5–8). Coloration is consistent among species of *Rhyncholepta*: reddish brown, with a large, impunctate pale spot on each basal angle of the scutellum and another pale, impunctate area along each anterolateral margin of the pronotum. This color pattern is not seen in any other chlorocorine species. A few species in other genera may have small pale spots or areas but these are usually confined to the hemelytra. *Rhyncholepta* can be distinguished from *Arvelius* by the unarmed abdominal base (spined in *Arvelius*) and the less developed mesosternal carinae (much more elevated in *Arvelius*). The apices of the mandibular plates are acute to spinose (with lateral margins of the mandibular plates relatively straight) in *Arvelius*, *Chlorocoris* (except the subgenusMonochrocerus), *Chloropepla*, *Fecelia* [acute only in *F.minor* (Vollenhoven, 1868)], *Loxa*, and *Mayrinia*. The apices of mandibular plates are narrowly rounded only in *Rhyncholepta* and ChlorocorissubgenusMonochrocerus; they are broadly rounded in *Eludocoris* (the head is broad apically, not subtriangular). Both *Chloropepla* and *Eludocoris* have elongate, apically acuminate metathoracic scent gland peritremes that separate those genera from all other chlorocorine genera, including *Rhyncholepta*. Furthermore, *Chlorocoris* and some species of *Chloropepla* lack the apical tooth on each femur that is present in all other chlorocorine genera.

Males of *Rhyncholepta* lack parameres in the genitalia. Besides *Rhyncholepta*, this condition is also known in four South American genera, *Luridocimex* Grazia, Fernandes & Schwertner, 1998, *Stysiana* Grazia, Fernandes & Schwertner, 1999 (both Carpocorini), *Patanius* Rolston, 1987 (unplaced in a tribe), and one still undescribed genus. None of these genera possess the characters found in the Chlorocorini (Rolston 1987; Grazia et al. 1998, 1999; Rider et al. 2018).

#### Etymology.

The generic name is composed of the Ancient Greek words ρύγχος (*rhýnchos*, = snout, muzzle, beak) and λεπτός (*leptós*, = thin), referring to the slender rostrum of the species. The gender is feminine, as it is evident from its ending -*a* and original combination with the adjective *grandicallosus* (-*a*, -*um*) given by Bergroth (1911) in its feminine form *grandicallosa*.

#### Bionomics.

Based on label data and Joe Eger’s and Roland Lupoli’s field experience, most of the specimens were collected by various types of light traps (UV, mercury vapor, metal halide, black, GemLight and Polyvie). GemLight and Polyvie traps are automatic light traps with visible light from LED, blue, pink, white or green; SEAG (= Société Entomologique Antilles-Guyane) is performing year-round surveys of insects in French Guiana using those traps (R Lupoli, pers. comm.). Almost all of the traps were exposed to fairly dense forest or adjacent to such a forest, except in Macouria, where one specimen was collected in the littoral secondary forest and one in the savanna. They have never been seen when collecting by hand catching, sweeping, or beating the vegetation during the day or night by JE Eger (pers. observ.) or R Lupoli (pers. comm.). One specimen was collected by flight intercept trap at Matiti, French Guiana, but there were no specimens of *Rhyncholepta* collected by glass interception traps operated by the SEAG during 3–4 years (R Lupoli, pers. comm.).

**Figures 94–97.**
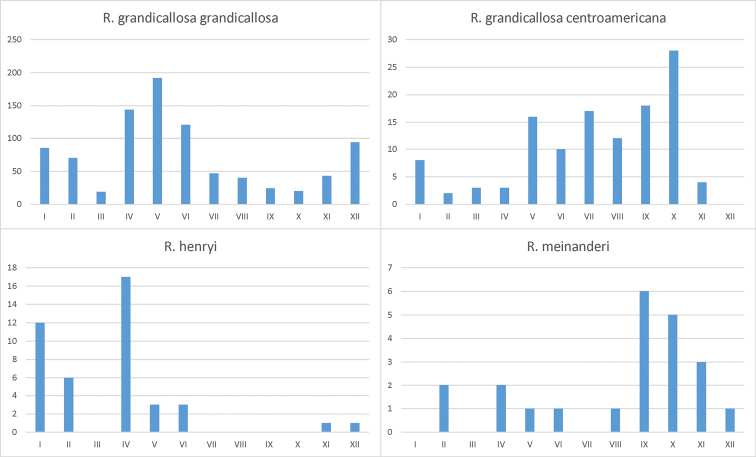
Annual distribution of collected *Rhyncholepta* specimens. **94***Rh.grandicallosagrandicallosa* Bergroth, 1911 (899 specimens with dates analysed) **95***Rh.grandicallosacentroamericana* subsp. n. (121 specimens) **96***Rh.henryi* sp. n. (43 specimens) **97***Rh.meinanderi* Becker & Grazia-Vieira, 1971 (22 specimens).

**Figures 98–100.**
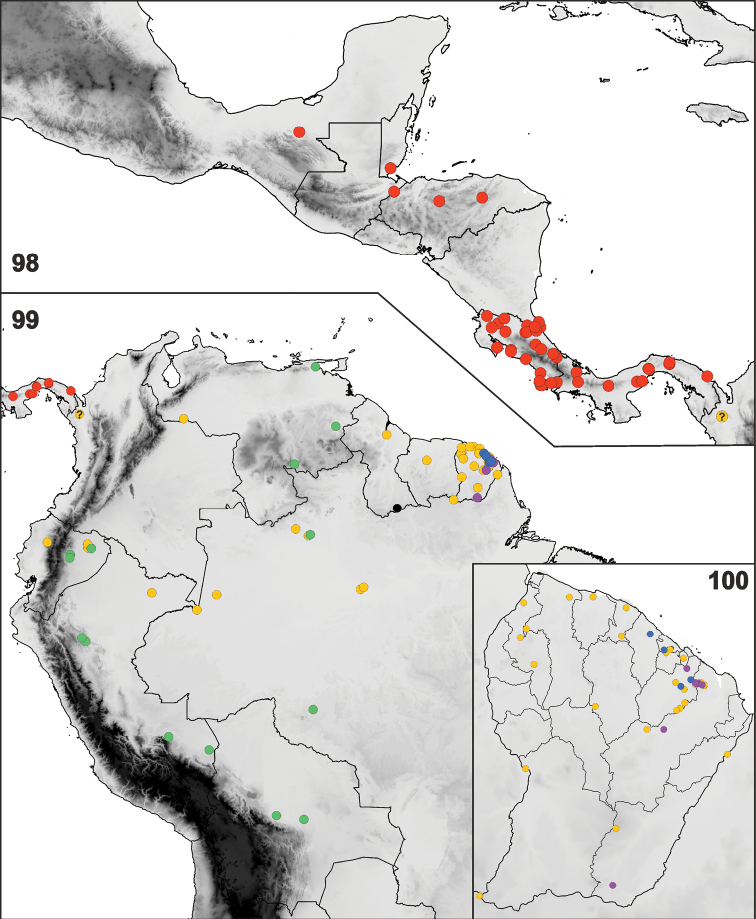
Distribution maps of *Rhyncholepta* species-group taxa. **98** Central America **99** South America **100** French Guiana. Color symbols: *Rhyncholeptagrandicallosagrandicallosa* Bergroth, 1911 (orange circles), *Rh.grandicallosacentroamericana* subsp. n. (red circles), *Rh.henryi* sp. n. (blue circles), syntopic occurrence of *Rh.g.grandicallosa* and *Rh.henryi* (purple circles), *Rh.meinanderi* Becker & Grazia-Vieira, 1971 (green circles), *Rh.wheeleri* sp. n. (black circle). Record of *Rh.grandicallosa* from Colombia with uncertain subspecies identity is marked by "?".

Collecting dates of the specimens examined indicate that species of *Rhyncholepta* are found year round, although distinct peaks might occur (Figs 94–98, especially in the case of *Rh.grandicallosagrandicallosa*).

#### Distribution

(Figs 98–100). The genus currently includes four species, one of them subdivided into two subspecies, distributed in the Neotropical Region from southern Mexico (Chiapas) to Bolivia and northwestern Brazil (Amazonas, Rondônia).

#### Species composition and delimitation.

Our examination of 1125 specimens revealed five more or less distinct morphotypes based almost exclusively on structure of the male genital capsule and especially the hypandrium (expanded portion of ventral rim). The five morphotypes may be grouped by morphological similarity as ((*grandicallosagrandicallosa* + *grandicallosacentroamericana*) (*henryi* (*meinanderi* + *wheeleri*))).

*Rhyncholeptagrandicallosa* differs from all three species of the *Rh.meinanderi* species-group by the following main characters: i) Genital capsule in ventral view with ventral rim apically bilobed, with small, shallow, V-shaped notch medially, hypandrial projections not visible in this view (Figs 30–35). ii) Anterior hypandrial projections large, lobe-like, apically rounded (Figs 42–47: ap, 72–74: ap). iii) Lateral hypandrial projections long, directed anteriad and golf-club shaped (Figs 54–59: lp). iv) Posterior hypandrial projections short, spinose, situated more laterally, directed ventrally (Figs 42–47: pp), not visible in ventral and dorsal views (Figs 30–35, 54–59). v) Phallus with aedeagus strongly S-shaped apically (Figure 82: ae). Within the morphotype of *Rh.grandicallosa*, the variability in shape of the anterior and lateral hypandrial projections enables two subtle, but stable, subtypes to be recognized. Differences between these subtypes are smaller than those between any two morphotypes/species of the *Rh.meinanderi* species-group, and both of the subtypes represent strictly allopatric populations. We, therefore, decided to classify them as subspecies: *Rh.g.grandicallosa* in South America and *Rh.g.centroamericana* subsp. n. in Central America.

The three remaining morphotypes form a distinct group characterized by the following shared characters: i) Genital capsule in ventral view with ventral rim apically convex, truncate or concave but never bilobed; posterior hypandrial projections visible in this view (Figs 36–41). ii) Anterior hypandrial projections triangularly narrowing, spinose at apex in dorsal (Figs 48–53: ap) and dorso-posterolateral (most exposed) view (Figs 75–77: ap). iii) Lateral hypandrial projections short, directed laterad, not golf-club shaped (Figs 60–65: lp). iv) Posterior hypandrial projections longer, spinose or widely rounded apically, situated more ventrally, directed ventrally or laterally (Figs 42–47: pp), visible in ventral and dorsal views (Figs 30–35, 54–59). v) Phallus with aedeagus only slightly sinuate apically (Figs 79: ae, 82: ae).

These morphotypes represent three related species that form the *Rh.meinanderi* species-group defined by the characters mentioned above. *Ryncholeptahenryi* sp. n. differs from *Rh.meinanderi* and *Rh.wheeleri* sp. n. by its different shape and position of the posterior hypandrial projection (narrowly rounded apically, directed laterally; Figs 37, 49, 61, 75: pp), and by the prominent posterolateral angles of the genital capsule (a character shared with *Rh.grandicallosa*). *Rhyncholeptameinanderi* and *Rh.wheeleri* sp. n. share the short spinous, ventrally directed posterior hypandrial projection (Figs 39, 41, 51, 53, 63, 65, 76–77: pp) and obtusangulate angles of the genital capsule. Both species differ in the shape of the three hypandrial projections (see key below).

Although we cannot confirm that the morphological differences between morphotypes reflect their phylogenetic relationships, the genus *Rhyncholepta* might be an interesting model group for phylogenetic and phylogeographic analyses.

##### Key to the males of *Rhyncholepta*

**Table:** 

1	Genital capsule in ventral view with ventral rim apically bilobed, small shallow V-shaped notch medially, hypandrial projections not visible (Figs 30–35). Hypandrium in posterior view with anterior projections large, lobe-like (not spinose apically) (Figs 42–47: ap); projections in dorso-posterolateral (most exposed) view large and rounded apically (Figs 72–74: ap). In dorsal view, lateral hypandrial projections long, directed anteriad and golf-club shaped (Figs 54–59: lp). Phallus with aedeagus S-shaped apically (Figure 82: ae)	***Rh.grandicallosa* Bergroth, 1911, 2**
–	Genital capsule in ventral view with ventral rim apically slightly convex, truncate, or widely V-shaped concave but not bilobed with shallow V-shaped notch medially, posterior hypandrial projections visible in this view (Figs 36–41). Anterior hypandrial projections triangularly narrowing, apically spinose both in dorsal (Figs 48–53: ap) and dorso-posterolateral (most exposed) view (Figs 75–77: ap). In dorsal view, lateral hypandrial projections shorter, directed laterad, not golf-club shaped (Figs 60–65: lp). Phallus with aedeagus only slightly sinuate apically (Figs 79, 82: ae)	***Rh.meinanderi* species-group, 3**
2	Anterior hypandrial projections in posterior view with apices appearing rounded (Figs 42, 43: ap), larger in most exposed (dorso-posterolateral) view, lateral margins parallel-sided in middle, widely rounded apically (Figs 55: ap, and 72: ap). In dorsal view, apices of anterior hypandrial projections directed right upwards, median outline regularly convex (Figs 54, 55: ap); lateral hypandrial projections with "handles" approaching each other towards apices (Figs 54, 55: lp), each apically regularly (C- shaped) curved inwards (Figs 55: lp, 72: lp). Northern South America	***Rh.grandicallosagrandicallosa* Bergroth, 1911**
–	Anterior hypandrial projections in posterior view with apices appearing acute (Figs 44, 45: ap, 46, 47: ap), smaller, parabolic, in most exposed (dorso-posterolateral) view apex narrowly rounded (Figs 73, 74: ap). In dorsal view, apices of anterior hypandrial projections directed anterodorsally, median outline straight (Figs, 56, 57: ap, 58, 59: ap); lateral hypandrial projections with "handles" nearly parallel, suddenly (ca. in right angle) curved inward apically (Figs 57, 59: lp, 73, 74: lp). Central America	***Rh.grandicallosacentroamericana* subsp. n.**
3	Genital capsule with posterolateral angles prominent (Figs 36, 60) in ventral and dorsal view. Ventral rim in ventral view truncate apically (Figs 36–37), posterior hypandrial projections directed laterad, rounded apically, together with ventral rim forming broad T (Figs 36–37). Anterior hypandrial projections in most exposed (dorso-posterolateral) view with dorsal margin nearly straight (Figure 75: ap); posterior and lateral projection forming acute angle (Figure 75: a); posterior projection appearing straight, wide, broadly rounded apically (Figure 75: pp). French Guiana	***Rh.henryi* sp. n.**
–	Genital capsule in ventral and dorsal view with posterolateral angles obtusangulate, not prominent (Figs 38, 40, 62, 64). Ventral rim in ventral view apically convex (Figs 38–39) or concave (Figs 40–41), posterior hypandrial projections directed ventrally, apically spinose (Figs 39, 41, 50–53). Posterior hypandrial projections in most exposed (dorso-posterolateral) view narrowing towards acute apex (Figs 76, 77: pp)	**4**
4	Ventral rim in ventral view apically widely convex; posterior projections lateral on median projection (Figs 38–39). Hypandrium in posterior view with posterior projections short, spinose, curved, apices directed ventrally (Figs 50, 51: pp). Anterior hypandrial projections in most exposed (dorso-posterolateral) view with dorsal margin slightly convex, apically with sharp spine bent downwards (Figure 76: ap); bases of posterior and lateral projections approached together, forming acute angle (Figure 76: a). In dorsal view, anterior hypandrial projections in apical half narrow (Figs 62, 63: ap), posterior projections directed laterad (Figs 62, 63: pp). Northern South America.	***Rh.meinanderi* Becker & Grazia-Vieira, 1971**
–	Ventral rim in ventral view apically with wide M-shaped projection, shallow V-shaped incision medially; posterior hypandrial projections posterolateral (Figs 40–41). Hypandrium in posterior view with posterior projections very short, acute but not spinose, directed posterolaterad (Figs 52, 53: pp). Anterior hypandrial projections in most exposed (dorso-posterolateral) view with dorsal margin slightly concave, apically with sharp spine strongly curved downwards (Figure 77: ap); bases of posterior and lateral projections widely separated, both projections parallel (Figure 77: a). In dorsal view, anterior hypandrial projections wide (Figs 64, 65: ap); posterior projections visible as acute angles directed posterolaterad (Figure 65: ppb). Guyana	***Rh.wheeleri* sp. n.**

### Rhyncholepta grandicallosa grandicallosa

Taxon classificationAnimaliaORDOFAMILIA

Bergroth, 1911

Figs 1–4 , 9 , 11–12 , 14–18 , 29 , 30–31 , 42–43 , 54–55 , 66 , 72 , 78–80 , 90 , 94

Rhyncholepta grandicallosa Bergroth, 1911: 121–122. Syntype(s): ♀, French Guiana (MZHF).
Rhyncholepta grandicallosa : Bergroth (1914): 441 (list), Pl. XI: fig. 6 (habitus illustration); Becker and Grazia-Vieira (1971): 393–396, 398–399, figs 1–3, 6–7, 10, 12, 14, 16 (redescription, habitus and detailed morphological illustrations, inadvertent lectotype designation, distribution); Arnold (2011): 104 (record); Greve et al. (2013): 3, 5, 12: fig. 4L, 14, 16: fig. 64 (cladistic analysis, morphology of male genitalia); Castro- Huertas et al. (2015) [?]: 563 (record); Rider et al. (2018): 190: fig. 2.21 (habitus photo, distribution); Silva et al. (2018): 437 (checklist).
Rhyncholepta grandicalosa (incorrect subsequent spelling): Grazia (1984): 79 (record).
Rhyncholepta sp. [?]: Torres Gutiérrez (2005): 95: fig. 4.42, 109, 115 (record, habitus photo).

#### Type locality.

French Guiana (without further details) (Bergroth 1911). Designation of neotype would change the type locality as follows: French Guiana, Roura Commune, Route de Kaw, Camp Caimans, 4°34'09.8"N 52°13'05.5"W, 320 m a.s.l.

**Type material examined.** Lectotype (designated by Becker and Grazia-Vieira (1971: 396), by use of "holótipo"; requested to be supressed by Kment et al. in press) (Figs 3–4, 14): ♀, **FRENCH GUIANA**: "FRENCH / GUIANA [handwritten in black ink, white label] // Rhyncholepta / grandicallosa B. [handwritten in black ink, white label] // Mus. Zool. H:fors. / Spec. Typ. No [printed] 12397 / Rhyncholepta / grandicallosa [handwritten in black ink and pencil, white label with black marginal frame] // Mus.Zool. Helsinki / Loan No. / HE [printed] 1246 [handwritten in blue ink, yellow label] // Mus.Zool. Helsinki / Loan No. / HE [printed] 1985 [handwritten in blue ink, yellow label] // Mus.Zool. Helsinki / Loan No. / HE [printed] 1985 [handwritten in blue ink, yellow label]" (MZHF). The lectotype is pinned through scutellum, antennomeres IIa–IV of both antennae missing, left hind leg and right fore and middle legs missing.

Neotype (here suggested) (Figs 1, 15): ♂, **FRENCH GUIANA**: "GUYANE FR., Rt. De Kaw / Camp Caimans, 320 m a.s.l. / 04.5694N, 52.2182W / 11.-19.i.2016, S. MURZIN lgt. [printed, white label] // COLLECTIO / NATIONAL MUSEUM / Praha, Czech Republic [printed, white label] // ♂ [printed, white label] // NEOTYPUS / *RHYNCHOLEPTA* / *G. grandicallosa* / Bergroth, 1911 / des. Kment, Eger, Rider 2017 [printed, red label]" (NMPC). The candidate neotype is card-mounted, with detached genital capsule glued on a separate small piece of card.

#### Additional material examined (males and associated females).

**BRAZIL: Amazonas**: Barcelos, Rio Aracá, boca do Rio Curuduri, 00°05'50"N 63°17'22"W, light trap, 15.–18.vi.2010, 1 ♂, C. Schwertner lgt., C. Schwertner and J. Grazia det. (INPA); Barcelos, Rio Aracá, comunidade Bacuquara, 00°09'17"N 63°10'35"W, light trap, 12.–14.vi.2010, 1 ♂, C. Schwertner lgt., C. Schwertner and J. Grazia det. (INPA); Barcelos, Rio Padauari, comunidade Ararão/Ararinha, 00°30'18"N 64°03'30"W, light trap, 4.–7.vi.2010, 1 ♂, C. Schwertner lgt., C. Schwertner and J. Grazia det. (INPA); Ipixuna, Rio Gregório, comunidade Lago Grande no Seringal do Recreio, 07°10'11.7"S 70°49'10.3"W, light trap, 17.–23.v.2011, 2 ♂♂ 3 ♀♀, C. Schwertner lgt., C. Schwertner and J. Grazia det. (INPA). – **ECUADOR: Napo Province**: Misahualli, 600 m a.s.l., 10.ix.1996, 1 ♂, D. Robacker lgt. (DBTC); Puerto Mis[a]hualli env., 1650–1900 ft [= 503–582 m a.s.l.], 1°2'49.2"S, 77°39'49.2"W, Mercury vapor and Ultraviolet lights, 6.–19.ix.1998, 2 ♂♂ 2 ♀♀, J. E. Eger lgt. (JEEC). **Orellana Province**: Cerca Pompeya, Yasuni NP, 00°38–40'S 76°22–27'W, 280 m a.s.l., 1.–11.x.2002, 3 ♂♂, D. Robacker lgt. (DBTC); Yasuni Research Station, ca. 40 km SE Limonccha, 3.–6.iv.2001, 1 ♂ 1 ♀, P. J. Landolt lgt. (JEEC). **Santo Domingo de los Tsáchilas Province**: Tinalandia [ca. 0.297304°S, 79.051773°W], 9.–16.vii.1980, 1 ♂, H. V. Weems, Jr. lgt. (FSCA). – **FRENCH GUIANA: Cayenne Arrondissement: Iracoubo Commune**: 16 km W Iracoubo, 5.49°N 53.31°W, 1.–2.i.2018, 1 ♂, S. Murzin lgt. (NMPC). **Macouria Commune**: Matiti, Za Wayabo, Flight Intercept Trap, 1.–31.iii.2013, 1 ♂ 1 ♀, J. L. Giuglaris lgt. (JEEC). **Montsinéry-Tonnegrande Commune**: 8 km W of Risquetout, 4°55.097'N 52°33.121'W, 45 m a.s.l., MV Light, 15.iv.2007, 1 ♂, D. G. Hall and J. E. Eger lgt. (FSCA). **Régina Commune**: 41 km SE Roura on Kaw Rd., 4°32.214'N 52°07.420'W, 272 m a.s.l., MV Light, 8.xii.2002, 4 ♂♂ 8 ♀♀, J. E. Eger lgt. (4 ♂♂ 4 ♀♀ FSCA, 4 ♀♀ JEEC); Route de Kaw, 4.5461°N 52.1221°W, 220 m a.s.l., 19.–23.xii.2015, 1 ♂, S. Murzin lgt. (ZJPC). **Roura Commune**: 14 km E of N2 on road to Dégrad Corréze, 4°29.964'N, 52°20.260'W, 108 m a.s.l., MV Light, 6.xii.2002, 2 ♂♂ 10 ♀♀, J. E. Eger lgt. (2 ♂♂ 5 ♀♀ FSCA, 5 ♀♀ JEEC); 15 km W of N2 on Belizon Rd., 6.–7.xii.2002, 1 ♂ 3 ♀♀, J. L. Giuglaris lgt. (FSCA); 17 km W of N2 on Belizon Rd., 4°17.825'N 52°22.812'W, 94 m a.s.l., MV Light, 3.xii.2002, 3 ♂♂ 1 ♀, J. E. Eger lgt. (2 ♂♂ FSCA, 1 ♂ 1 ♀ JEEC); 27 km SE Roura on Kaw Rd., 4°34.116'N 52°12.614'W, 308 m a.s.l., MV Light, 12.–20.xi.2009, 4 ♂♂ 8 ♀♀, L. Pöllumea and O. Maasikas lgt. (FSCA); the same locality, 12.–14.xii.2009, 2 ♂♂ 2 ♀♀, L. Pöllumea and O. Maasikas lgt. (1 ♂ 1 ♀ FSCA, 1 ♂ 1 ♀ JEEC); the same locality, 5.ii.2010, 2 ♂♂, J. E. Eger lgt. (FSCA); 28 km SE Roura on Kaw Rd., 4°34.252'N, 52°12.797'W, 306 m a.sl., MV Light, 17.ii.2010, 2 ♂♂, J. E. Eger lgt. (FSCA); 32 km SE Roura on Kaw Rd., 4°33.612'N 52°11.350'W, 287 m a.s.l., MV Light, 15.ii.2010, 8 ♂♂ 3 ♀♀, J. E. Eger lgt. (7 ♂♂ 3 ♀♀ FSCA, 1 ♂ JEEC); 33 km SE Roura on Kaw Rd., 4°34.135'N, 52°11.150'W, 227 m a.s.l., MV Light, 1.xii.2002, 1 ♂ 1 ♀, J. E. Eger lgt. (FSCA); the same locality, MV Light, 26.xii.2002, 1 ♂ 5 ♀♀, F. Goubert lgt. (1 ♂ 3 ♀♀ FSCA, 2 ♀♀ JEEC); the same locality, MV Light, 1.–2.vi.2005, 5 ♂♂ 7 ♀♀, J. E. Eger and M. T. Messenger lgt. (FSCA); the same locality, MV Light, 12.–13.iv.2007, 17 ♂♂ 22 ♀♀, D. G. Hall and J. E. Eger lgt. (FSCA); 38 km SE Roura on Kaw Rd., 4°34.214'N, 52°09.566'W, 256 m a.s.l., MV Light, 4.xii.2002, 5 ♂♂, J. E. Eger lgt. (4 ♂♂ FSCA, 1 ♂ JEEC); the same locality, 25.xii.2002, 1 ♂, F. Goubert lgt. (FSCA); Amazone Nature Lodge env., 30 km SE Roura on Kaw Rd., 4°33.570'N, 52°12.433'W, 300 m a.s.l., UV Light Trap, 2.–8.vi.2005, 2 ♂♂ 1 ♀, J. E. Eger and M. T. Messenger lgt. (FSCA); the same locality, UV Light Trap, 10.–18.iv.2007, 3 ♂♂ 1 ♀♀, D. G. Hall and J. E. Eger lgt. (FSCA); the same locality, UV Light Trap, 5.–19.ii.2010, 3 ♂♂ 2 ♀♀, J. E. Eger lgt. (3 ♂♂ 1 ♀ FSCA, 1 ♀ JEEC); the same locality, MV Lights, 4.–15.i.2016, 11 ♂♂ 11 ♀♀, J. Eger, R. Morris and J. Wappes lgt. (JEEC); 1 km S Amazone Nature Lodge, 30 km SE Roura on Kaw Rd., 4°32.961'N, 52°12.830'W, 288 m a.s.l., 3.–4.vi.2005, 1 ♂ 3 ♀♀, J. E. Eger and M. T. Messenger lgt. (FSCA); Cacao env., 150 m a.s.l., 4.572°N 52.427°W, 2.–4.i.2018, 1 ♂ 3 ♀♀, S. Murzin lgt. (1 ♂ 1 ♀ NMPC, 1 ♀ HNHM, 1 ♀ NHMW); Entomotech Lodge, 30 km SE Roura on Kaw Rd.N, 4°33.570'N 52°12.433'W, 300 m a.s.l., MV Light, 1.–12.xii.2002, 6 ♂♂ 7 ♀♀, J. E. Eger lgt. (4 ♂♂ 3 ♀♀ FSCA, 2 ♂♂ 4 ♀♀ JEEC); the same locality, MV Light, xi.2004–ii.2005, 4 ♂♂ 3 ♀♀, 2.ii.2005, 2 ♂♂, F. Goubert lgt. (FSCA); Highway D6 to Kaw, 33.5 km SE of Roura [ca. 4°32'47"N 52°08'41"W], 10.ii.1986, 1 ♂ 2 ♀♀, G. Tavakilian lgt. (DARC); Kaw Road km 18, 26.viii.1995, 1 ♂, J. E. Wappes lgt. (DBTC); Route de Kaw, Caiman Camp env., 4.i.2007, 1 ♂, M. Snížek lgt. (ZJPC; labeled as paraneotype); Route de Kaw, Camp Caimans, 4.5694°N 52.2182°W, 320 m a.s.l., 11.–19.i.2016, 3 ♂♂ 1 ♀, S. Murzin lgt. (2 ♂♂ 1 ♀ NMPC, 1 ♂ BMNH, males labeled as paraneotypes); 20.–31.i.2016, 2 ♂♂ 5 ♀♀, S. Murzin lgt. (1 ♂ 2 ♀♀ NMPC, 2 ♂♂ 4 ♀♀ ZJPC; males labeled as paraneotypes); 1.–3.ii.2016, 3 ♂♂ 1 ♀, S. Murzin lgt. (1 ♂ 1 ♀ NMPC, 2 ♀♀ ZJPC, male labeled as paraneotypes); Camp Caiman, 4.569°N 52.218°W, 260 m a.s.l., 8.–31.i.2018, 2 ♂♂ 1 ♀, S. Murzin lgt. (1 ♂ 1 ♀ NMPC, 1 ♂ MMBC; males labeled as paraneotypes). **Sinnamary Commune**: Sinnamary [ca. 5.374628°N 52.955196°W], Route de St. Elie, 14.i.2007, 1 ♂, M. Snížek lgt. (ZJPC). **Saint-Georges Commune**: Pied Saut, Oyapok River [= north bank of the Oyapock River, ca. 12 km upstream of Saint-Georges, at the foot of the first rapids on this, 3°48'30"N 51°52'30"W – see Ingels et al. (2012)], 1 ♂, S. M. Klages lgt., C. M. Acc. 6111, D. B. Thomas det. (DBTC). **Saint-Laurent-du-Maroni Arrondissement: Mana Commune**: Rte. d’Apatou (Chutes Voltaire), 5.15°N 54.023°W, 6.–31.xii.2017, 3 ♂♂ 1 ♀, S. Murzin lgt. (1 ♂ 1 ♀ NMPC, 1 ♂ HNHM, 1 ♂ NHMW). **Saint-Laurent-du-Maroni Commune**: Camp Voltaire, 5.0530°N 54.0881°W, 60 m a.s.l., 25.–31.xii.2015, 3 ♂♂ 2 ♀♀, S. Murzin lgt. (1 ♂ NMPC, 2 ♂♂ 2 ♀♀ ZJPC). – **PERU: Loreto Province**: 80 km NE Iquitos [ca. 3.341878°S 72.741851°W], Explorama Lodge, 1 km from Amazon R.[iver] on R.[iver] Yanamono, at light, 25.–28.viii.1992, 1 ♂, J. Castner, P. Skelley et al. lgt. (JEEC).

#### Material examined (tentatively identified females).

**ECUADOR: Orellana Province**: Yasuni Research Station, 250 m a.s.l., 0°38'S, 76°36'W, 17.–31.x.1998, 1 ♀, B. K. Dozier lgt. (JEEC). **Sucumbíos Province**: Limoncocha on Rio Napo [ca. 0°24'24"S 76°37'15"W], 13.v.1974, 1 ♀, 19.i.1974, 1 ♀, B. A. Drummond, III lgt. (JEEC). – **FRENCH GUIANA: Cayenne Arrondissement: Régina Commune**: 21 km SE Roura on Kaw Rd., 4°36.115'N, 52°15.972'W, MV Light, 6.–7.ii.2010, 3 ♀♀, J. E. Eger lgt. (2 ♀♀ FSCA, 1 ♀ JEEC); 33 km SE Roura on Kaw Rd., 4°34.135'N, 52°11.150'W, 227 m a.s.l., MV Light, 7.xii.2002, 2 ♀♀, J. E. Eger lgt. (FSCA); Entomotech Lodge, 30 km SE Roura on Kaw Rd.N, 4°33.570'S 52°12.433'W, 300 m a.s.l., MV Light, 5.xii.2004, 1 ♀, 6.xii.2004, 1 ♀, 9.xii.2004, 1 ♀, F. Goubert lgt. (FSCA). **Not identified**: Montagne Tortua, 26.viii.1981, 1 ♀, G. Tavakilian lgt. (DARC). – **GUYANA**: Essequibo R.[iver], Moraballi Creek [Moraballi Creek about 3 km above junction with Essequibo River, 6°11'N – see Davis and Richards (1933) / 6°12'16.9"N 58°33'51.6"W], 4.ix.1929, 1 ♀, Oxf. Univ. Expedn., B.M. 1929-485 (BMNH); New River, iii.–v.1938, 1 ♀, viii.1938, 1 ♀, C. A. Hudson lgt., Brit. Mus. 1939–370 (BMNH). – **SURINAME**: Raleigh Falls [4°40'N 56°09'W], 25.–27.vii.1975, 1 ♀, L. H. Rolston lgt. (DARC).

#### Material identified by Roland Lupoli (deposited in RLFF).

**FRENCH GUIANA: Cayenne Arrondissement: Camopi Commune**: Itoupé [Mt.; 3°01'N 53°04'W], 600–800 m a.s.l., Light Trap, 19.xi.–1.xii.2014, 5 spec., SEAG [= Société Entomologique Antilles-Guyane] lgt. **Macouria Commune**: Forêt littorale de Maya, Polyvie (Blue LED) Trap, 12.xii.2015, 1 spec., SEAG lgt.; Savane Lambert [ca. 4°53'26"N 52°31'46"W], Polyvie (Blue LED) Trap, 9.vii.2016, 1 spec., SEAG lgt. **Matoury Commune**: La Désirée, Polyvie Trap (Blue LED), 8.vi.2014, 4 spec., 20.ix.2014, 1 spec., SEAG lgt. **Saint-Elie Commune**: Inselberg Haute-Koursibo [4°18'59"N 53°17'10"W], Light Trap, 3.iii.2013, 3 spec., SEAG lgt.; the same locality, Polyvie (Blue LED) Trap, 5.iii.2013, 1 spec., 26.x.2013, 1 spec., 2.xi.2013, 1 spec., SEAG lgt.; Réserve Naturelle de la Trinité [ca. 4°04'18"N 52°33'18"W], Zone Bénitier, Light Trap, 9.x.2010, 1 spec., 7.–8. and 10.xi.2013, 5 spec., SEAG lgt. **Régina Commune**: Piste Bélizon, km 20, Light Trap, 26.viii.2003, 1 spec., 21.xii.2003, 1 spec., R. Lupoli lgt.; Piste Bélizon, km 4, 8.v.2004, 5 spec., R. Lupoli lgt.; RN2, km 136, Light Trap, 8.iv.2014, 1 spec., SEAG lgt.; Nouragues [ca. 4°04'18"N 52°43'57"W], Saut Pararé, Light Trap, 23.vii.2009, 1 spec., 22.ii.2010, 2 spec., SEAG lgt.; Nouragues, Inselberg, Automatic Light Trap, 13.x.2012, 6 spec., SEAG lgt. **Roura Commune**: Route de Kaw, km 36–38, 9.ii.1993, 1 spec., Lecourt lgt.; the same locality, Light Trap. 12.ix.1998, 1 spec., 23.ix.2000, 2 spec., 24.viii.2003, 1 spec., 6.v.2004, 1 spec., 10.v.2004, 3 spec., Lupoli lgt.; Montagne des Chevaux RN2 km 22 [ca. 4.7216°N 52.3073°W], 23.v.2009, 1 spec., SEAG lgt.; the same locality, Automatic Light Trap, 20.vi.2009, 1 spec., 14.v.2010, 1 spec., 5.ii.2012, 1 spec., SEAG lgt.; the same locality, GemLight Trap, 20.v.2012, 1 spec., SEAG lgt.; the same locality, Polyswing Trap, 8.vii.2012, 1 spec., SEAG lgt.; the same locality, Automatic Light Trap, 21.x.2012, 1 spec., SEAG lgt.; the same locality, GemLight Trap, 9.xii.2012, 3 spec., SEAG lgt.; the same locality, Polyvie Trap (Blue LED), 16.xii.2012, 2 spec., 24.xii.2012, 6 spec., 9.i.2013, 3 spec., 13.i.2013, 5 spec., 27.i.2013, 9 spec., 4.ii.2013, 2 spec., 11.ii.2013, 14 spec., 16.ii.2013, 5 spec., 24.ii.2013, 2 spec., SEAG lgt.; the same locality, GemLight Trap, 4.iii.2013, 1 spec., SEAG lgt.; the same locality, Polyswing and GemLight Traps, 24.iii.2013, 2 spec., SEAG lgt.; the same locality, Polyvie (Blue LED) and GemLight Traps, 6.iv.2013, 10 spec., 13.iv.2013, 37 spec., 20.iv.2013, 18 spec., 27.iv.2013, 17 spec., 4.v.2013, 48 spec., 13.v.2013, 42 spec., 19.v.2013, 40 spec., 25.v.2013, 22 spec., SEAG lgt.; the same locality, Polyvie Trap (Blue LED), 1.vi.2013, 29 spec., SEAG lgt.; the same locality, Polyvie (Blue LED) and GemLight Traps, 8.vi.2013, 19 spec., 15.vi.2013, 27 spec., 22.vi.2013, 15 spec., 29.vi.2013, 1 spec., 6.vii.2013, 13 spec., 13.vii.2013, 21 spec., 20.vii.2013, 3 spec., 27.vii.2013, 4 spec., 3.viii.2013, 3 spec., 10.viii.2013, 5 spec., 17.viii.2013, 3 spec., 24.viii.2013, 1 spec., 31.viii.2013, 1 spec., 7.ix.2013, 1 spec., 14.ix.2013, 2 spec., 21.ix.2013, 1 spec., 5.x.2013, 3 spec., 19.x.2013, 1 spec., 3.xi.2013, 1 spec., SEAG lgt.; the same locality, Polyvie Trap (Blue LED), 30.xi.2013, 1 spec., 14.xii.2013, 1 spec., 28.xii.2013, 1 spec., 4.i.2014, 3 spec., SEAG lgt.; the same locality, GemLight Trap, 11.i.2014, 1 spec., SEAG lgt.; the same locality, Polyvie Trap (Blue LED), 18.i.2014, 1 spec., 25.i.2014, 2 spec., 1.ii.2014, 5 spec., 15.ii.2014, 1 spec., 29.iii.2014, 7 spec., SEAG lgt.; the same locality, Polyvie Trap (Rose and Blue LED), 5.iv.2014, 2 spec., SEAG lgt.; the same locality, GemLight Trap, 19.iv.2014, 2 spec., 27.iv.2014, 2 spec., SEAG lgt.; the same locality, Polyvie Trap (Rose and Blue LED), 27.iv.2014, 4 spec., SEAG lgt.; the same locality, Polyvie (Blue LED) and GemLight Traps, 3.v.2014, 2 spec., SEAG lgt.; the same locality, Polyvie Trap (Rose and Blue LED), 17.v.2014, 4 spec., 31.v.2014, 5 spec., SEAG lgt.; the same locality, GemLight Trap, 7.vi.2014, 1 spec., SEAG lgt.; the same locality, Polyvie Trap (Blue LED), 26.vii.2014, 1 spec., 2.viii.2014, 2 spec., 9.viii.2014, 5 spec., 21.viii.2014, 13 spec., 30.viii.2014, 1 spec., SEAG lgt.; the same locality, Polyvie Trap (Rose LED), 20.ix.2014, 1 spec., 27.ix.2014, 1 spec., SEAG lgt. **Sinnamary Commune**: Route Barrage Petit Saut km 21–22 [ca. 5°04'14"N 53°00'21"W], Light Trap, 11.ii.2002, 3 spec., R. Lupoli lgt.; the same locality, Light Trap, 29.iv.2002, 4 spec., 23.v.2003, 1 spec., 4.vi.2003, 1 spec., all Bout lgt.; the same locality, Light Trap, 12.x.2004, 1 spec., Lupoli lgt. **Saint-Laurent-du-Maroni Arrondissement: Mana Commune**: Laussat ouest [ca. 5°29'16"N 53°33'46"W], Light Trap, 14.v.2010, 1 spec., Lamarre lgt. **Maripasoula Commune**: DZ rivière Coulé coulé, Light Trap, 22.x.2004, 1 spec., Champenois lgt.; Massif du Mitaraka [ca. 2°17'29"N 54°31'18"W], Light Traps, 23.ii.–25.iii.2015, 49 spec., MNHN lgt. **Saül Commune**: Belvédère [ca. 2.41°N 53.1°W], Light Trap, 13.iii.2013, 1 spec., SEAG lgt.; the same locality, Polyvie (Blue LED) Trap, 1.ix.2015, 2 spec., 15.ix. 2015, 6 spec., 13.xi.2015, 4 spec., 27.xi.2015, 2 spec., 11.xii.2015, 1 spec., 8.i.2016, 14 spec., 22.ii.2016, 1 spec., 9.iii.2016, 1 spec., 31.v.2016, 8 spec., SEAG lgt. **St-Laurent du Maroni Commune**: Village Espérance [ca. 5°25'39"N 54°03'04"W], Polyvie Trap (Blue LED), 1.iv.2014, 1 spec., 15.v.2014, 2 spec., SEAG lgt.; Sommet Massif Lucifer [ca. 4°45'57"N 53°56'26"W], Light Trap, 25.x.2014, 1 spec., SEAG lgt.

#### Diagnosis.

Coloration, structure of head, thorax and pregenital abdomen, and vestiture as in other species of the genus (see redescription of *Rhyncholepta* above) except the following characters:

*Apex of scutellum* with anteapical black V-shaped stripe usually reduced to small black spot at lateral margin at anterior end of apical V-shaped callosity (Figs 14–17). However, two examined males (♂ [Figure 18], French Guiana, Camp Caimans, 20.–31.i.2016, NMPC; ♂, French Guiana, Pied Saud, DBTC), have the black V complete. Apical callosity large, branches of the V short, forming ca. one third, less frequently one half, of length, tip of scutellum therefore with rather large triangular callosity (Figs 14–18).

*Male genitalia.* Genital capsule in ventral view distinctly constricted lateroapically (Figs 30: arrow, 54: arrow), posterolateral angles prominent, ca. rectangular (Figs 30, 54); dorsal wall at base of posterolateral angles deeply impressed (Figs 54: arrow, 66). Ventral rim in ventral view apically bilobed, with shallow V-shaped notch medially (Figs 30–31); hypandrial projections not visible in ventral view (Figs 30–31). Hypandrium in posterior view with pair of large-lobe like anterior projections, apices appearing rounded (Figs 42, 43: ap) and very short, pointed posterior projections directed posterolaterad (Figs 42, 43: pp); lateral projections not visible in ventral view but placement of their attachment apparent as small convexity laterally on anterior projections (Figure 43: lpb). Anterior hypandrial projection in most exposed (dorso-posterolateral) view larger than in *Rh.grandicallosacentroamericana*, with lateral margins parallel-sided in middle, broadly rounded apically (Figure 72: ap). In dorsal view, apices of anterior hypandrial projections directed straight upwards, their median outline regularly convex (Figs 54, 55: ap; straight in *Rh.g.centroamericana*); lateral hypandrial projections long, golf-club shaped, their "handles" approaching apically (Figs 54, 55: lp; nearly parallel in *Rh.g.centroamericana*), apically regularly (C-shaped) curved inwards (Figs 55: lp, 72: lp). *Phallus* (Figs 78–80; described and illustrated in detail by Becker and Grazia-Vieira 1971: figs 6, 7, 10): Basal plates U-shaped. Phallotheca cylindrical, curved dorsally at right angles at apex. Conjunctiva variably sclerotized ventrally, with pair of laminar sclerites flanking distal region of aedeagus, but not reaching phallotreme (Figure 79: cjs); dorsally with strong expansion giving conjunctiva sacculiform appearance. Aedegaus (= vesica) elongate, S-shaped, covered with conjunctiva except at apex (Becker and Grazia-Vieira 1971: fig. 10; in Figure 79: ae the S-shaped sinuation less prominent due to overmaceration in KOH).

*Female genitalia*. Posterior edges of laterotergites VIII abruptly attenuated apically, as long as or slightly more prominent posteriad compared with laterotergites IX (Figure 90). Internal female genitalia described in detail by Becker and Grazia-Vieira (1971: figs 14, 16).

*Measurements.* See Table 1. Measurements of candidate neotype (in mm): Body length 12.07, body length to segment VII 10.85, head length 2.35, head width 2.25, interocular width 1.03, length of antennomeres: I – 0.83, IIa – 1.23, IIb – 2.50, III – 3.43, IV – 2.94, pronotum length 2.55, pronotum width 8.24, scutellum length 4.41, scutellum width 3.53.

#### Differential diagnosis.

See characters in the key above. Most specimens of this subspecies differ from *Rh.henryi* sp. n. by the incomplete black V-shaped band anteapically on scutellum; however, this character does not work for all specimens.

#### Etymology.

The species name is a composed Latin adjective, *grandicallosus* (-*a*, -*um*), meaning "bearing large callosities."

#### Bionomics.

Specimens mainly were collected by various types of light traps (UV light, mercury vapor light, GemLight and Polyvie traps) in dense forests or adjacent to such a forest, except in Macouria, where one specimen was collected in the littoral secondary forest and one in the savanna. This species has never been collected by hand catching, sweeping, or beating the vegetation during the day or night. One specimen was collected by flight intercept trap at Matiti, French Guiana (JE Eger, pers. observ.; R Lupoli, pers. comm.). Collection dates of specimens examined indicate that *Rh.g.grandicallosa* occurs year round, with a distinct peak in April–June (Figure 94) (Becker and Grazia-Vieira 1971, Castro Huertas et al. 2005, this paper).

#### Distribution

(Figs 99–100). Brazil: Amazonas (Becker and Grazia-Vieira 1971, Arnold 2011, Silva et al. 2018); ?Colombia: Chocó (Torres Gutiérrez 2005, as *Rhyncholepta* sp.; Castro-Huertas et al. 2015); French Guiana (Becker and Grazia-Vieira 1971); Guyana (new country record); Peru (new country record); Suriname (new country record); Venezuela (Becker and Grazia-Vieira 1971, Grazia 1984).

Records from Colombia, Guyana, and Suriname require confirmation based on males. The subspecific identity of *Rh.grandicallosa* population from Chocó, Colombia requires revision.

#### Comments.

Bergroth (1911) described the species based on the female sex, but did not indicate the number of specimens (syntypes) examined, though the fact that a single measurement and not a range was given for the body length (‘Long. ♀ (sine membr. [= without membrane]) 11 mm) suggests that he had only one specimen. Bergroth (1914: fig. 6) provided a color painting of an adult. Neither of the Bergroth’s two papers mentioned the depository of the type(s).

Pirán (1956: 29, 35: fig. 1) illustrated the male genitalia of a specimen from Bolivia and designated the specimen as the allotype. The designation of a male allotype by Pirán (1956) is an invalid action without nomenclatural consequence because his specimen was not a part of the original type series (see ICZN 1999: Article 72). Furthermore, his illustration more closely conforms to *Rh.meinanderi* as described by Becker and Grazia-Vieira (1971).

Becker and Grazia-Vieira (1971: 396) referred to a female holotype from French Guiana, deposited in the collection of the MZHF. We consider the action of Becker and Grazia-Vieira (1971: 396) as a valid lectotype designation under Article 74.6 (ICZN 1999) because the term holotype was used explicitly for the only existing (syn)type specimen. Becker and Grazia-Vieira (1971) distinguished two species of the genus *Rhyncholepta*. They interpreted *Rh.grandicallosa* based on their examination of the lectotype and described its male and a new species, *Rh.meinanderi*, which differs from *Rh.grandicallosa* in structure of the male and female genitalia (Becker and Grazia-Vieira 1971: figs 2, 3 versus 4, 5 and 12 versus 13).

Our revision of the genus *Rhyncholepta*, however, reveals five taxa that are indistinguishable based on coloration, structure of body and pregenital abdomen, vestiture, and morphometric characters (see Table 1). The most promising external character, the development of the black V-shaped anteapical band on the scutellum and its apical V-shaped callosity, might help in identifying specimens of *Rh.grandicallosagrandicallosa* versus *Rh.henryi*, but we found two males of *Rh.g.grandicallosa* from French Guiana with a complete V-shaped band on the scutellum, as in *Rh.henryi*. This character also varies widely in *Rh.grandicallosacentroamericana*. The painting by Bergroth (1914), depicting almost certainly the lectotype of *Rh.grandicallosa*, shows an apparent black V-shaped band anteapically on the scutellum. The black V-shaped band, however, is not well delimited in recent photographs of the lectotype (see Figure 5), which might be attributed to inaccuracy of the painting or fading of the specimen’s coloration during a century of preservation.

The female external genitalia allow *Rh.meinanderi* to be distinguished from both subspecies of *Rh.grandicallosa* and *Rh.henryi* sp. n., but those taxa cannot be reliably separated based on this character (Figs 93 versus 90–92). Also, the internal female genitalia did not provide suitable identification characters. Moreover, the female of *Rh.wheeleri* sp. n., the taxon probably most closely related to *Rh.meinanderi*, remains unknown.

The structure of the male genital capsule and, to a lesser extent, of the phallus thus remain the only reliable characters for identifying species of *Rhyncholepta*. The presence of two sympatric taxa in French Guiana, the type locality of *Rh.grandicallosa*, required reconsideration of the identity of this taxon. Careful examination of the photographs of the lectotype provided by MZHF confirmed that, in the absence of a reliable character allowing identification of female *Rh.grandicallosa* and *Rh.henryi*, the lectotype is not sufficient to determine the specific identity of *Rh.grandicallosa*. We decided to follow Article 75.5 of ICZN (1999) by petitioning the International Commission on Zoological Nomenclature to suppress the existing non-informative lectotype and replace it with a male neotype. Herein we suggest a suitable male neotype, which is properly documented to fulfill all requirements of Article 75.3 of ICZN (1999) and conserves the identity of *Rh.grandicallosa* sensu Becker and Grazia-Vieira (1971) and all subsequent authors. The case is being submitted to the ICZN simultaneously with this paper (Kment et al., submitted).

### Rhyncholepta grandicallosa centroamericana subsp. n.

Taxon classificationAnimaliaORDOFAMILIA

http://zoobank.org/6B0F453F-819C-4360-BC13-9E87ED75901A

Figs 5 , 10 , 13 , 19–22 , 26–28 , 32–35 , 44–47 , 56–59 , 67–68 , 73–74 , 81–83 , 91 , 95

Rhyncholepta grandicallosa : Becker and Grazia-Vieira (1971): 396 (partim, records from Panama); Froeschner (1999): 185 (checklist); Arismendi (2002): 31 (distribution); Arismendi and Thomas (2003): 223, 230 (distribution, records); Cambra et al. (2018): 13, 17: Figure 37 (list, photo).

#### Type locality.

Panama, Panamá Province, El Lano Cartí Road, km 8–11, 1100' [= 335 m a.s.l.], ca. 9°17'N 78°58'W.

#### Type material.

Holotype: ♂, "PAN. Panama Prv / El Lano Carti Rd k / 8–11 24 May–2 June / 1992 1100' JE Wappes [printed, white label] // DBT [printed, white label] // ♂ [printed, white label] // HOLOTYPUS / *RHYNCHOLEPTA* / *grandicallosa* subsp. / *CENTROAMERICANA* nov. / det. Kment, Eger, Rider 2017 [printed, red label]" (DBTC → USNM). The holotype is pinned through scutellum, left distiflagellum is missing.

Paratypes: **MEXICO: Chiapas**: Palenque [17°29'15"N 92°02'47"W], MV Light, 19.viii.1990, 1 ♂, P. J. Landolt lgt. (JEEC). – **BELIZE**: British Honduras, 5.iv.1937, 1 ♂, no collector, J. Grazia-Vieira 1973 det. (DARC); British Honduras, Rio Grande, viii.1935, 1 ♀, J. J. White lgt., B.M. 1935-597, *Rhyncholeptagrandicallosa* det. Ruckes 1961 (BMNH). – **GUATEMALA: Izabal Province**: 30 km SE of Morales, Finca Firmeza [15.379°N 88.695°W], at light, 7.–22.vii.2008, 4 ♂♂ 2 ♀♀, F. Skillman, C. and L. O’Brien lgt. (FSCA); Izabal, road up to Firmeza, 30 km SE Morales, 22.vii.2008. 1 ♂, C. W. and L. B. O’Brien and F. Skillman lgt. (JEEC); D. Izabel, Firmeza, 30 km SE Morales, at UV and metal halide light, 23.vii.2008, 1 ♀, C. W. and L. B. O’Brien and F. Skillman lgt. (JEEC). – **COSTA RICA: Alajuela Province**: 20 km S Upala [ca. 10°43'11"N 85°01'06"W], 11.–21.vi.1991, 1 ♀, F. D. Parker lgt. (DBTC); Ca. 15 km SW Volcán Arenal, Arenal Vista Lodge [ca. 10°25'47"N 84°45'40"W], at mercury vapor and black light, 13.–15.viii.1995, 1 ♂ 1 ♀, J. E. Eger coll. [Costa Rica Collecting Permit No. 00113412] (1 ♂ JEEC, 1 ♀ INBIO); Caño Negro [ca. 10°53'35"N 84°47'45"W], R.N.V.S., 20 m a.s.l., 5.–28.ii.1995, 1 ♂, K. F. Flores lgt., L_N_319100_450200 #4424 (INBIO); Finca Monte Sele [ca. 10.57°N 85.25°W], 750 m a.s.l., ix.1994, 2 ♂♂, C. Moraga lgt., L N 326200_379900 #3204 (INBIO). **Cartago Province**: Monumento Nacional Guayabo [ca. 9°58'16"N 83°41'27"W], Turrialba, 1100 m a.s.l., ix.1994, 1 ♂, G. Fonseca lgt., L N 217200_570300 #3202 (INBIO); Paso Marcos env., 9°48.71'N 83°29.85'W, 2450' [= 747 m a.s.l.], 1 ♂, D. Thomas, D. Robacker and W. Warfield lgt. (DBTC). **Guanacaste Province**: Est. Pitilla, 9 km S Sta. Cecilia, P. N. Guanacaste, xi.1988, 1 ♂, C. Chaves and M. Espinosa lgt., L-N 330200, 380200 (INBIO); Est. Pitilla, 9 km S Sta. Cecilia, P. N. Guanacaste, i.–ii.1990, 1 ♂, GNP Biod. Survey, L-N 330200, 380200 (INBIO); Est. Pitilla, 9 km S Sta. Cecilia [ca. 10°58'48"N 85°24'48"W], P. N. Guanacaste, x.1992, 1 ♂, C. Moraga lgt., L-N 330200, 380200 (INBIO); Hacienda El Oro, 450–500 m a.s.l., vii.1996, 1 ♂, A. Masis, M. M. Chavarria, C. Moraga, P. Ríos, de Luz [= light trap], L_N_332600_377400 #45263 (INBIO). **Heredia Province**: Est. El Ceibo, Braulio Carrillo N. P., 400–600 m a.s.l., v.1990, 1 ♀, C. Chaves lgt., L-N-527700, 256500 (INBIO); Est. Magsasay [ca. 10°24'19"N 84°03'18"W], P.N. Braulio Carrillo, 200 m a.s.l., v.1991, 1 ♀, A. Fernández lgt., L-N-264600, 531000 (INBIO); Finca la Selva Verde, 12 km S. Puerto Viejo, 500 ft [= 152 m a.s.l.], 23.–26.ix.1986, 1 ♂ 1 ♀, J. E. Eger lgt. (JEEC); La Selva Biological Station, 3 km S Pto. Viejo, 10°26'N 84°01'W, 26.vii.1992, 2 ♂♂, H. A. Hespenheide lgt., La Selva Project (DARC); Los Arbolitos [ca. 10°38'47"N 83°59'36"W], 30 m a.s.l., 20.–27.iii.1993, 2 ♂♂, F. Araya lgt., L N 536100_291400 #1952 (1 ♂ FSCA, 1 ♂ INBIO); near Puerto Viejo, La Selva Biological Station, 10°25'N 84°00'W, 179 ft [= 55 m a.s.l.], at light, 1.iii.2004, 1 ♂, C. R. Bartlett, J. Cryan and J. Urban lgt. (DARC). **Limón Province**: Amubri [ca. 9°31'34"N 82°57'08"W], 70 m a.s.l., 12.–31.x.1993, 1 ♂, G. Gallardo lgt., L S 385500_578000 #2407 (INBIO); Amubri, A. C. Amistad, 70 m a.s.l., 2.–20.ix.1993, 1 ♂, G. M. Gallardo lgt., L S 385500_578000 #2368 (INBIO); Cerro Tortuguero [ca. 10°35'02"N 83°31'38"W], P.N. Tortuguero, 100 m a.s.l., xi.1989, 1 ♂, J. Solano lgt., L-N 285000, 588000 (INBIO); Cerro Tortuguero, P.N. Tortuguero, 0–100 m a.s.l., v.1990, 1 ♀, J. Solano lgt., L-N 285000, 588000 (INBIO); Cerro Tortuguero, P.N. Tortuguero, 0–120 m a.s.l., vi.1991, 2 ♂♂, R. Delgado lgt., L-N 285000, 588000 (INBIO); Est. Cuatro Esquinas [ca. 10°27'23"N 83°40'19"W], P. N. Tortuguero, 0 m a.s.l., 27.iii.–29.iv.1992, 2 ♂♂, D. Garcia lgt., L-N 280000, 590500 (INBIO); Est. Hitoy Cerere, R. Cerere, Res. Biol. Hitoy [ca. 9°38'50"N 83°04'15"W], 100 m a.s.l., 28.–12.[sic!].iv.1992, 1 ♂, E. Lopez lgt., L-N 184200, 643300 (INBIO); Río Sardinas, R.N.F.S. Barra del Colorado [ca. 10°46'07"N 83°35'08"W], 10 m a.s.l., 14.x.1992, 1 ♂, F. Araya lgt., L N 291500_564700 (INBIO); Río Sardinas, R.N.F.S. Barra del Colorado, 10 m a.s.l., 12.–30.ix.1993, 4 ♂♂ 4 ♀♀, F. Araya lgt., L N 291500_564700 #2355 (2 ♂♂ 2 ♀♀ INBIO, 1 ♂ BMNH, 1 ♂ 1 ♀ NHMW, 1 ♀ NMPC); Río Sardinas, R.N.F.S. Barra del Colorado, 10 m a.s.l., 11.–19.x.1993, 1 ♀, F. Araya lgt., L N 291500_564700 #2398 (INBIO); Río Sardinas, R.N.F.S. Barra del Colorado, 10 m a.s.l., 2.–12.i.1994, 1 ♂, F. Araya lgt., L N 291500_564700 #2552 (INBIO); R.V.S., Barra del Colorado, Camino a Linda Vista, 98 m a.s.l., 19.viii.2004, 1 ♂, B. Gamboa, W. Porras, D. Briceno, M. Moraga and Y. Cárdenas lgt., light trap, LN 284965 568835//77943 (INBIO); Sardinas, Barra del Colorado, 15 m a.s.l., 27.iii.–3.iv.1995, 2 ♂♂, F. Araya lgt., L N 291900 565900 #4414 (INBIO); Sector Cerro Cocori, Fca de E. Rojas [E. Rojas' farm; 10.60°N, 83.72°W], xi.1990, 1 ♂, E. Rojas lgt., L-N-286000, 567500 (INBIO). **Puntarenas Province**: Esquipulas de Tasazu, 27 km NE of Quepos, 27.v.2003, 2 ♂♂ 1 ♀, J. C. Burne lgt. (DBTC); Est. Agujas [ca. 8.537998°N 83.471381°W], 300 m a.s.l., night collecting, 25.–28.x.1995, 1 ♂, A. Azofeifa lgt., L_S_276750_526550 #6373 (INBIO); Est. Quebrada Bonita, R. B. Carara [ca. 9°47'02"N 84°34'02"W], 80 m a.s.l., xi.1994, 1 ♂, J. C. Sobrio lgt., L N 194500_469850 #3290 (INBIO); Est. Sirena [ca. 8°32'37"N 83°31'18"W], P. N. Corcovado, 0–100 m a.s.l., x.1989, 1 ♂, G. Fonseca lgt., L-S-270500, 508300 (INBIO → FSCA); x.1989, 1 ♂, G. Fonseca lgt., L-S-270500, 508300 (FSCA); the same locality, x.1989, 1 ♂, C. Saborio lgt., L-S-270500, 508300 (INBIO → FSCA); Est. Sirena, P. N. Corcovado, 1–100 m a.s.l., vi.1990, 1 ♂, G. Maass lgt., L-S-270500, 508300 (INBIO); Est. Sirena, P. N. Corcovado, 1–100 m a.s.l., vi.1990, 1 ♀, N. Obando lgt., L-S-270500, 508300 (INBIO); Est. Sirena, P. N. Corcovado, 1–100 m a.s.l., v.1994, 1 ♂, G. Fonseca lgt., L S 270500_508300 #2899 (INBIO); Sirena, Corcovado Nat. Pk., Osa Peninsula, 14.viii.1980, 1 ♂, H. Janzen and W. Hallwachs lgt., INBIO, CRI001 715796 (INBIO → FSCA); Golfo Dulce, P.N. Corcovado, Est. Agujas [ca. 8.537998°N 83.471381°W], 200–300 m a.s.l., 10.–12.x.2007, 1 ♂, J. A. Azofeifa lgt., Tp. Luz [= light trap], L_S_276750_526550 #92557 (INBIO); Lepanto, Montaña Grande, Estac. Karen Mogensen [ca. 9°52'14"N 85°03'30"W], 320 m a.s.l., 25.–30.ix.2003, 1 ♂ 1 ♀, Y. Cardenas lgt., Tp. de Luz [= light trap], L_N_205600_420300 #75455 (INBIO); near Villa Neily [ca. 8°38'47"N 82°56'44"W], 5.–11.viii.1963, 1 ♂, C. L. Hogue lgt. (DARC); Osa, Ciudad P[uer]to Cortés [ca. 8°57'52"N 83°31'33"W], Cuesta del Burro, 680 m a.s.l., 6.vi.2005, 1 ♂, J. Montero, B. Gamboa, J. Gutiérrez, M. Moraga, J. Azofeifa, Y. Cárdenas and J. Mata, Tp. Luz. [= light trap], L_S_330629_517352 #83476 (INBIO); Osa Peninsula, Sirena, Corcovado Nat. Pk., 13.viii.1980, 2 ♂♂, D. H. Janzen and W. Hallwachs lgt. (INBIO); R. Priv. Karen Mogensen, Alred. Estación, 350 m a.s.l., 6.vii.2003, 1 ♀, M. A. Zumbado and W. Porras lgt., Tp. Luz. Mercurio [= mercury vapor light], L N 205600 420300 #74586 (INBIO); R.V.S. Río Piro, Golfito [ca. 8°37'09"N 83°08'40"W], Finca Catalino, 200 m a.s.l., 16.ix.2004, 1 ♀, Y. Cardenas, D. Briseño and B. Gamboa, Luz. [= light trap], L_S_264550_535590 #78215 (INBIO); Rancho Quemado [ca. 8°40'55"N 83°33'38"W], Peninsula de Osa, x.1990, 6 ♂♂ 13 ♀♀, F. Quesada lgt., L-S-292500, 511000 (1 ♂ 5 ♀♀ FSCA, 2 ♂♂ 5 ♀♀ INBIO, 1 ♀ BMNH, 1 ♂ 1 ♀ HNHM, 2 ♂♂ 1 ♀ NMPC); Rancho Quemado, Pen.[insula de] Osa, x.–xi.1990, 1 ♀, B. Apu lgt., L-S-292500, 511000 (INBIO); Rancho Quemado, Pen.[insula de] Osa, i.1991, 3 ♂♂ 2 ♀♀, F. Quesada lgt., L-S-292500, 511000 (INBIO); Rancho Quemado, Pen.[insula de] Osa, iv.1991, 1 ♂, J. C. Saborio lgt., L-S-292500, 511000 (INBIO); Sirena, Corcovado Nat. Pk., Osa Penin.[sula], 5.–11.i.1981, 1 ♀, D. H. Janzen and W. Hallwachs lgt. (INBIO). **San José Province**: Est. Bijagual [ca. 9°43'43"N 84°34'09"W] , Res. Biol. Carara, 500 m a.s.l., ix.1990, 1 ♂, G. Varela lgt., L-N-192250, 474760 (INBIO). – **PANAMA: Bocas del Toro Province**: 12 km W Chiriqui Grande [ca. 8°59'32"N 82°15'13"W], 10.–13.v.1999, 2 ♂♂, Morris and Wappes lgt. (JEEC). **Chiriquí Province**: Finca La Suiza, 5.3 km N Los Planes, 8°39'N, 82°12'W, 4500' [= 1372 m a.s.l.], 26.–30.v.1995, 1 ♂, B. Ratcliffe and M. Jameson lgt. (DBTC). **Coclé Province**: Rio Indio Lodge, N El Valle, 8°39'46.7"N, 80°7'7.9"W, 575 m a.s.l., 23.–27.ii.2012, 1 ♀, J. B. Heppner lgt. (FSCA). **Colón Province**: Pipeline Road, km 0, 9°7'19"N, 79°42'53"W, beating of forest vegetation along strips for boat navigation signs, 31.viii.2010, 1 ♂ 2 ♀♀, L. Sekerka lgt. (MMBC). **Panamá Province**: Campana [= Cerro Campana], 2785 ft [= 849 m a.s.l.], 8°40.920'N 79°55.731'W, 28.v.–9.vi.2008, 1 ♀, D. C. Robacker lgt. (DBTC); 10–12 km N El Llano, 3.–8.vi.1986, 1 ♀, E. Giesbert lgt. (FSCA); El Llano–Carti Rd. [= road], km 10–13 [ca. 9°17'37"N 78°58'43"W], 3.–7.vi.1984, 1 ♀, R. L. Penrose, F. T. Hovore and P. H. Sullivan lgt. (DBTC); K[m] 8–13 El Llano–Carti Rd. [= road], 10.–13.v.1996, 2 ♂♂ 1 ♀, Wappes, Huether and Morris lgt. (1 ♂ 1 ♀ DBTC, 1 ♂ JEEC); PN Chagres, Cerro Jeffe, 9°14.3' N 79°24.1' W, 700–950 m a.s.l., lower montane forest; individual collecting, 18.–19.viii.2017, 2 ♂♂, M. Seidel and L. Sekerka lgt. (NMPC). **San Blas Province**: Nusagandi [9.3489°N, 78.966°W], 18.–20.v.1993, UV light, 3 ♂♂, E. Riley lgt. (2 ♂♂ DARC, 1 ♂ DBTC); Nusagandi, at light, 250–350 m a.s.l., 26.vii.1995, 1 ♂ 3 ♀♀, C. W. and L. B. O’Brien lgt. (JEEC); San Blas, Punta Eseoses [= Escocés; 8.85°N 77.6333°W], Lt. Trap, ii.–iii.1979, 1 ♂ 1 ♀, Caroline Ash lgt., H. D. Engleman det. as *Rhyncholeptagrandicallosa* (JEEC). **Veraguas Province**: Santa Fé env., Cascada Alto de Piedra, 8°31.0'N 81°07.3'W, 830 m a.s.l., lower montane forest, individual collecting, 12.ix.2017, 1 ♂, J. Hájek lgt. (NMPC). – All the paratypes are bearing the following identification label: "PARATYPUS / *RHYNCHOLEPTA* / *grandicallosa* subsp. / *CENTROAMERICANA* nov. / det. Kment, Eger, Rider 2017 [printed, yellow label]".

#### Diagnosis.

Coloration, structure of head, thorax and pregenital abdomen, and vestiture as in other species of the genus (see redescription of *Rhyncholepta* above) except the following characters.

*Apex of scutellum* with anteapical black V-shaped stripe usually well developed, wide (Figure 20) to narrow (Figs 21–22), less frequently reduced to small black spot on each lateral margin at anterior end of apical V-shaped callosity (Figure 19); all color forms can be syntopic (e.g. Costa Rica, Rio Sárdinas, INBIO). Apical callosity V-shaped, robust, branches of V forming ca. one third to one half of width, tip of scutellum with distinct triangular callosity (Figs 19–22).

*Male genitalia.* Genital capsule in ventral view more or less constricted lateroapically (Figs 32: arrow, 34: arrow, 56: arrow, 58: arrow), posterolateral angles prominent, ca. rectangular (Figs 32, 34, 56, 58); dorsal wall at base of posterolateral angles shallowly to deeply impressed (56: arrow, 58: arrow, 67–68). Ventral rim in ventral view bilobed apically, with shallow V-shaped notch medially (Figs 32–35); hypandrial projections not visible in ventral view (Figs 32–35). Hypandrium in posterior view with pair of large lobe-like anterior projections, apices appearing acute (Figs 44, 45: ap, 46, 47: ap) and short-pointed posterior projections directed posterolaterad (Figs 45: pp, 47: pp); lateral projections not visible in ventral view but site of their attachment apparent as obtuse angle laterally on anterior projections (Figure 45: lbp, 47: lbp). Anterior hypandrial projections in most exposed (dorso-posterolateral) view smaller than in *Rh.grandicallosagrandicallosa*, parabolic, with narrowly rounded apex (Figs 73: ap, 74: ap). In dorsal view, apices of anterior hypandrial projections directed anterodorsally, median outline straight (Figs 56, 57: ap, 58, 59: ap; in *Rh.g.grandicallosa* convex); lateral hypandrial projections long (appearing longer than in *Rh.g.grandicallosa*), golf-club "handles" nearly parallel, suddenly curved inwards (ca. in right angle) apically (Figs 57: lp, 59: lp, 73: lp, 74: lp). *Phallus* (Figs 81–83) conjunctival sclerites (Figure 82: cjs) and aedeagus strongly S-shaped as in *Rh.g.grandicallosa* (Figure 79; Becker and Grazia-Vieira 1971: fig. 10).

*Female genitalia*. Posterior edges of laterotergites VIII posteriorly as long as or slightly more prominent compared with laterotergites IX (Figure 91) (within variation of *Rh.g.grandicallosa*).

*Measurements.* Table 1. Measurements of holotype (in mm): Body length 13.19, body length to segment VII 11.64, head length 2.56, head width 2.41, interocular width 1.11, length of antennomeres: I – 0.84, IIa – 1.43, IIb – 2.73, III – 3.44, IV – 2.73, pronotum length 2.71, pronotum width 7.88, scutellum length 4.65, scutellum width 3.83.

#### Variability.

Besides the usual variation in coloration and structure within *Rhyncholepta* species, we observed some variability of *Rh.g.centroamericana* in the structure of the genital capsule and hypandrium, as illustrated in two males. One is from Panama, Pipeline Road (Figs 32–33, 44–45, 56–57, 67, 73; namely genital capsule slightly constricted posterolaterally [Figs 32, 56], anterior hypandrial projection longer, with apex more prominent, and median margin slightly less concave medially [Figs 45: ap, 57: ap, 73: ap]). The second male is from Costa Rica, Rancho Quemado (Figs 34–35, 46–47, 58–59, 68, 74; namely genital capsule hardly constricted posterolaterally [Figs 34, 58], anterior hypandrial projection shorter, with apex less prominent, and median margin slightly more concave medially [Figs 47: ap, 59: ap, 74: ap]). The posterolateral constriction of the genital capsule in the specimen from Panama, Pipeline Road (Figs 32, 56: arrow) is somewhat intermediate between the typical *Rh.g.grandicallosa* (see Figs 30, 54: arrow) and the population from Costa Rica, Rancho Quemado (Figs 34, 58: arrow).

#### Etymology.

The subspecies name is a Latin adjective *centroamericanus* (-*a*, -*um*) referring to its Central American distribution.

#### Bionomics.

Most specimens were collected by various types of light traps (UV light, mercury vapor light, metal halide light, black light) in or adjacent to dense forests. According to JE Eger, it has never been collected by hand catching, sweeping, or beating vegetation during the day or night. In Panama, three specimens were collected by "beating of forest vegetation along strips for boat navigation signs" (J Sekerka, pers. comm.) and one in "lower montane forest, [by] individual collecting" (J Hájek, pers. comm.) but without detailed information. Collection dates indicate that *Rh.g.centroamericana* occurs year round, though most specimens were collected between May and October (Figure 95) (Becker and Grazia-Vieira 1971, Arismendi and Thomas 2003; present paper).

#### Distribution

(Figure 98). Mexico: Chiapas (new country record); Belize (new country record); Guatemala (new country record); Honduras (Arismendi 2002, Arismendi and Thomas 2003, both as *Rh.grandicallosa*); Costa Rica (Arismendi 2002, as *Rh.grandicallosa*); Panama (Becker and Grazia-Vieira 1971, Froeschner 1999, Arismendi 2002, Cambra et al. 2018, all as *Rh.grandicallosa*).

The subspecific identity of *Rh.grandicallosa* population from Chocó, Colombia requires revision.

### Rhyncholepta henryi sp. n.

Taxon classificationAnimaliaORDOFAMILIA

http://zoobank.org/DF08B4F4-0B65-4A2C-98FD-C299E534174B

Figs 6 , 23 , 36–37 , 48–49 , 60–61 , 69 , 75 , 84–86 , 92 , 96

#### Type locality.

French Guiana, Roura Commune, Route de Kaw, Camp Caimans, 4°34'09.8"N 52°13'05.5"W, 320 m a.s.l.

#### Type material.

Holotype: ♂ (Figs 6, 23, 36–37, 48–49, 60–61, 69, 75), "GUYANE FR., Rt. de Kaw / Camp Caimans, 320 m a.s.l. / 04.5694N, 52.2182W / 11.-19.i.2016, S. MURZIN lgt. [printed, white label] // COLLECTIO / NATIONAL MUSEUM / Praha, Czech Republic [printed, white label] // ♂ [printed, white label] // HOLOTYPUS / *RHYNCHOLEPTA* / *HENRYI* / sp. nov. / det. Kment, Eger, Rider 2017 [printed, red label]" (NMPC). The holotype is card-mounted, with the detached left distiflagellum glued on the same piece of card and the detached genital capsule and abdominal segment VIII glued separately on a small piece of card.

Paratypes: **FRENCH GUIANA: Cayenne Arrondissement: Kourou Commune**: Montagne des Signes near Kourou [ca. 5.091899°N 52.699481°W], collected at mercury vapor light, 3.vi.1986, 2 ♂♂, E. C. Riley and D. A. Rider lgt. (DARC). **Roura Commune**: 27 km SE Roura on Kaw Rd., 4°34.116'N, 52°12.614'W, MV Light, 5.ii.2010, 1 ♂, J. E. Eger lgt. (JEEC); 32 km SE Roura on Kaw Rd., 4°33.612'N, 52°11.350'W, 287 m a.s.l., MV Light, 15.ii.2010, 1 ♂, J. E. Eger lgt. (JEEC); 33 km SE Roura on Kaw Rd., 4°34.135'N, 52°11.150'W, 227 m a.s.l., MV Light, 12.–13.iv.2007, 6 ♂♂, D. G. Hall and J. E. Eger lgt. (JEEC); Amazone Nature Lodge, 30 km SE Roura on Kaw Rd., 4°33.570'N, 52°12.433'W, 300 m a.s.l., UV Light, 10.–18.iv.2007, 1 ♂, D. G. Hall and J. E. Eger lgt. (JEEC); the same locality, MV Lights, 4.–15.i.2016, 4 ♂♂, J. Eger, R. Morris and J. Wappes lgt. (JEEC); Camp Caiman, 4.569°N 52.218°W, 260 m a.s.l., 8.–31.i.2018, 3 ♂♂, S. Murzin lgt. (NMPC); Entomotech Lodge, 30 km SE Roura on Kaw Rd., 4°33.570'N 52°12.433'W, 300 m a.s.l., MV Light, 17.xi.2004, 1 ♂, xi.2004–ii.2005, 2 ♂♂, 17.i.2005, 1 ♂, F. Goubert lgt. (JEEC); Highway N2 to Regina, 45 km S of Cayenne [ca. 4°31'53"N 52°22'20"W], collected at mercury vapor light, 31.v.1986, 2 ♂♂, E. G. Riley and D. A. Rider lgt. (DARC); Montagne des Chevaux RN2 km 22 [ca. 4.7216°N 52.3073°W], automatic light trap (white LED), 3.i.2013, 1 ♂, SEAG leg. (MNHN). **Saint-Laurent-du-Maroni Arrondissement: Mana Commune**: Réserve Trinité [ca. 4°04'18"N 52°33'18"W], Drop Zone Aya, UV light trap, 30.v.2012, 1 ♂, SEAG leg. (RLFF). **Saül Commune**: Belvédère [ca. 2.41°N 53.1°W], automatic light trap (blue LED), 5.vii.2017, 1 ♂, SEAG leg. (RLFF). – All the paratypes are bearing the following identification label: "PARATYPUS / *RHYNCHOLEPTA* / *HENRYI* / sp. nov. / det. Kment, Eger, Rider 2017 [printed, yellow label]".

#### Additional material examined

**(females tentatively identified as *Rh.henryi*). FRENCH GUIANA: Cayenne Arrondissement: Kourou Commune**: Montagne des Signes near Kourou [ca. 5.091899°N 52.699481°W], collected at mercury vapor light, 3.vi.1986, 1 ♀, E. C. Riley and D. A. Rider lgt. (DARC). **Montsinéry-Tonnegrande Commune**: 8 km W of Risquetout, 4°55.097'N 52°33.121'W, 45 m a.s.l., MV Light, 15.iv.2007, 3 ♀♀, D. G. Hall and J. E. Eger lgt. (JEEC). **Roura Commune**: 21 km SE Roura on Kaw Rd., 4°36.115'N, 52°15.972'W, MV Light, 6.–7.ii.2010, 1 ♀, J. E. Eger lgt. (JEEC); 28 km SE Roura on Kaw Rd., 4°34.252'N, 52°12.797'W, 306 m a.s.l., MV Light, 17.ii.2010, 1 ♀, J. E. Eger lgt. (JEEC); 32 km SE Roura on Kaw Rd., 4°33.612'N, 52°11.350'W, 287 m a.s.l., MV Light, 15.ii.2010, 1 ♀, J. E. Eger lgt. (JEEC); 33 km SE Roura on Kaw Rd., 4°34.135'N, 52°11.150'W, 227 m a.s.l., MV Light, 12.–13.iv.2007, 7 ♀♀, D. G. Hall and J. E. Eger lgt. (JEEC); Amazone Nature Lodge, 30 km SE Roura on Kaw Rd., 4°33.570'N, 52°12.433'W, 300 m a.s.l., UV Light, 4.–15.i.2016, 1 ♀, J. Eger, R. Morris and J. Wappes lgt. (JEEC); Camp Caiman, 4.569°N 52.218°W, 260 m a.s.l., 8.–31.i.2018, 1 ♀, S. Murzin lgt. (NMPC); Entomotech Lodge, 30 km SE Roura on Kaw Rd., 4°33.570'N 52°12.433'W, 300 m a.s.l., MV Light, 1.–12.xii.2002, 1 ♀, J. E. Eger lgt. (DARC); Highway D6 to Kaw, 33.5 km SE of Roura [ca. 4°32'47"N 52°08'41"W], 10.ii.1986, 1 ♀, G. Tavakilian lgt. (DARC).

#### Diagnosis.

Coloration, structure of head, thorax and pregenital abdomen, and vestiture as in other species of the genus (see redescription of *Rhyncholepta* above) except for the following characters.

*Apex of scutellum* with anteapical black V-shaped stripe well developed (Figure 23). Apical V-shaped callosity thin, narrowly delineating margins of scutellum apex, branches forming more than half of length, tip of scutellum lacking conspicuous triangular callosity (Figure 23).

*Male genitalia.* Genital capsule in ventral view only slightly constricted lateroapically, posterolateral angles prominent (Figs 36, 60); dorsal wall at base of posterolateral angles shallowly impressed (Figs 60, 69). Ventral rim in ventral view truncate apically, slightly notched medially (Figs 36–37), posterior hypandrial projections visible and caudal, together with ventral rim forming wide T (Figs 36–37); bases of lateral projections also visible (Figure 37). Hypandrium in posterior view with three pairs of projections: posterior ones caudal, short, flat, narrowly rounded apically with small rounded projection anteapically on posterior margin (Figs 48–49); lateral projections very narrow, directed posterolaterad, apices spinose, curved upwards (Figs 48–49); anterior projections steeply sloping downwards, appearing narrowly triangular in dorsal view, acutangulate apically (Figs 48–49). Anterior hypandrial projections in most exposed (dorso-posterolateral) view appearing nearly triangular with dorsal margin nearly straight, apically with sharp, straight, flattened spine (Figure 75: ap); posterior and lateral projections forming acute angle (Figure 75: a); posterior projection appearing straight, wide, widely rounded apically in this view (Figure 75: pp); lateral projection narrowing, shortly bent downwards apically (Figure 75: lp). Anterior hypandrial projections narrowing anteriad towards apices in dorsal view, apices straight, each forming large claw-like spine curved downwards (Figs 60, 61: ap), posterior projections short, flat, apically rounded (Figs 60, 61: pp); lateral projections directed laterad, each narrowing to sharp spine, curved anteriad apically (Figs 60, 61: lp). *Phallus* (Figs 84–86) with basal conjunctival sclerite drop-shaped (Figure 85: cjs) and aedeagus only slightly sinuate apically (Figure 85: ae), very similar to that of *Rh.meinanderi*.

*Female genitalia*. Posterior edges of laterotergites VIII suddenly attenuated apically, posteriorly about as long as laterotergites IX (Figure 92), indistinguishable from *Rh.grandicallosa*.

*Measurements.* Table 1. Measurements of holotype (in mm): Body length 12.39, body length to segment VII 10.91, head length 2.55, head width 2.27, interocular width 1.15, length of each antennomeres: I – 0.86, IIa – 1.34, IIb – 2.61, III – 3.46, IV – 2.95, pronotum length 2.47, pronotum width 8.05, scutellum length 4.37, scutellum width 3.62.

#### Differential diagnosis.

See above key. All specimens differing from the majority of specimens of *Rh.g.grandicallosa* by complete black V-shaped band anteapically on scutellum (character does not work for all specimens of *Rh.g.grandicallosa*).

#### Etymology.

We are pleased to dedicate the new species to our colleague, Thomas J. Henry, an excellent specialist in Heteroptera and curator of the USNM, on the occasion of his 70^th^ birthday. We much appreciate his generosity in helping visitors with literature or specimens in the USNM, including hosting colleagues in his home when they visited the collection.

#### Bionomics.

Most specimens were collected by various types of light traps (UV light, mercury vapor light, white and blue LED) in or adjacent to dense forests. This species has never been collected by hand catching, sweeping or beating vegetation during the day or night (JE Eger, pers. observ.). *Rhyncholeptahenryi* was found from November to February and from April to June, with most specimens collected in April (Figure 96).

#### Distribution

(Figs 99–100). French Guiana (present paper).

### Rhyncholepta meinanderi

Taxon classificationAnimaliaORDOFAMILIA

Becker & Grazia-Vieira, 1971

Figs 7 , 24 , 38–39 , 50–51 , 62–63 , 70 , 76 , 87–89 , 93 , 97

Rhyncholepta grandicallosa (misidentification): Pirán (1956): 29 (record, invalid allotype designation), 35: fig. 1 (line drawing of apex of male abdomen in ventral view).
Rhyncholepta meinanderi Becker & Grazia-Vieira, 1971: 394, 397–399, figs 4–5, 8–9, 11, 13, 15, 17 (description, illustrations of morphological details, distribution).
Rhyncholepta meinanderi : Grazia (1984): 79 (record); Arnold (2011): 104 (record).

#### Type locality.

Venezuela, Bolívar, Karnakuni, 450 m a.s.l., ca. 4°26'N 64°08'W.

#### Type material (not examined).

Holotype: ♂, "Kanarakuni, Bolivar, Venezuela, 450 m, 4-II-1967, F. Fernandez Y. and A. D. Ascoli col." (IZAV).

#### Material examined.

**BOLIVIA: Santa Cruz Department**: Prov.[incia] del Sara [ca. 17°02'16"S 63°32'47"W], C M Acc 5068, xi.1913, 1 ♀, Steinbach lgt. (DARC). – **BRAZIL: Amazonas**: Barcelos, Rio Aracá, comunidade Bacuquara, 00°09'17"N 63°10'35"W, 12.–14.vi.2010, 1 ♂, C. Schwertner lgt. and det. (INPA). **Rondônia**: 62 km SW Ariquemes near Fzda. [= farm] Rancho Grande [10°17'51"S 62°52'08"W], MV and Black Lights, 5.–17.x.1993, 3 ♂♂ 1 ♀, J. E. Eger lgt. (JEEC). 62 km SW Ariquemes near Fzda. Rancho Grande, BLT, 25.xi.1993, 1 ♀, 15.ix.1994, 1 ♀, 25.xi.1994, 1 ♀, U. Schmitz lgt. (JEEC). – **ECUADOR: Orellana Province**: Yasuni National Park, 00°40.478'S 76°23.866'W, 29.iv.2005, 1 ♂ 1 ♀, C. R. Bartlett, N. Nazdrowicz and D. Chang lgt. (DARC). **Napo Province**: Puerto Mis[a]hualli env., 1650–1900 ft [= 503–582 m a.s.l.], 1°2'49.2"S 77°39'49.2"W, Mercury vapor and Ultraviolet lights, 6.–19.ix.1998, 1 ♂ 4 ♀♀, J. E. Eger lgt. (JEEC). **Pastaza Province**: Arajuno env. [ca. 1°14'25"S 77°40'53"W], 3.–10.xii.2000, 1 ♂, V. Kabourek lgt. (ZJPC). – **PERU: San Martín Province**: Lejias, 59 km N. Tarapoto, 6°18.123'S 76°43.588'W, 1110 m a.s.l., 5.ii.2005, 1 ♂, D. B. Thomas, Warfield, R. Cave, D. Robacker and H. Panduro-Salas lgt. (DBTC). Moyabamba, vic. Ecologica "Rumipata", 6°04'32.0"S 76°58'07.5"W, 970 m a.s.l., MV and UV Light, 13.–18.x.2012, 1 ♂, J. E. Eger lgt. (JEEC). **Madre de Dios Province**: Rio Tambopata Res., 30 km (air) SW Pto. [= Puerto] Maldonado, 12°50'S 69°17'W, 290 m a.s.l., 2.v.1984, 1 ♀, Smithsonian Institution Canopy Fogging Project (02/03), T. L. Erwin et al. lgt. (USNM).

#### Diagnosis.

Coloration, structure of head, thorax and pregenital abdomen, and vestiture as in other species of the genus (see redescription of *Rhyncholepta* above) except the following characters.

*Apex of scutellum* with anteapical black V-shaped stripe on scutellum reduced to small black spot or single black puncture on each lateral margin at anterior end of the apical V-shaped callosity (Figure 24). Apical V-shaped callosity wide, its branches one third or less of its length, tip of scutellum with distinct triangular callosity (Figure 24).

*Male genitalia.* Genital capsule in ventral view slightly constricted lateroapically, posterolateral angles obtusangulate, not prominent (Figs 38, 62); dorsal wall at base of posterolateral angles shallowly impressed (Figs 62, 70). Ventral rim in ventral view broadly convex apically, posterior hypandrial projections lateral on projected portion of ventral rim, pointed and directed ventrally (Figs 38–39); only basal portions of lateral projections visible in this view (Figs 38–39). Hypandrium in posterior view with three pairs of projections: posterior ones caudal, short, spinose, curved, apices directed ventrally (Figs 50, 51: pp); lateral projections very narrow, directed posterolaterad, apices spinose, nearly straight (Figs 50, 51: lp); anterior projections steeply sloping downwards, appearing narrowly triangular, acutangulate apically, apices directed dorsolaterad (Figs 50, 51: ap). Anterior hypandrial projections in most exposed (dorso-posterolateral) view appearing roughly quadrangular, dorsal margins slightly convex, apically each with sharp spine bent downwards (Figure 76: ap); bases of posterior and lateral projections forming acute angle (Figure 76: a); posterior projection narrowing towards acute apex (Figure 76: pp); lateral projection narrowing, apically straight (Figure 76: lp). In dorsal view, anterior hypandrial projections narrowing triangularly in basal half, apical half narrow, parallel-sided, slightly divergent, apices acute (Figs 62, 63: ap); only bases of posterior projections visible (Figure 63: ppb); lateral projections directed laterad, each narrowing to sharp spine, apically curved slightly anteriad (Figure 63: lp). *Phallus* (Figs 87–89; described in detail by Becker and Grazia-Vieira 1971: figs 8, 9, 11) with basal conjunctival sclerite cylindrical (Figure 88: cjs), aedeagus only slightly sinuate apically (Figure 88: ae), very similar to that of *Rh.henryi*.

*Female genitalia* (Figure 93). Laterotergites VIII triangularly produced apically, posteriorly distinctly more prominent than posterior margins of laterotergites IX. Internal female genitalia described in detail by Becker and Grazia-Vieira (1971: figs 15, 17).

#### *Measurements.*

Table 1.

#### Differential diagnosis.

See above key. Females with laterotergites VIII triangularly produced apically, distinctly more produced posteriorly (Figure 93). From *Rh.henryi* sp. n. it also differs by reduced V-shaped black band anteapically on scutellum.

#### Etymology.

The species was dedicated to Dr. Martin Meinander, former curator of Hemiptera in the Finnish Museum of Natural History, Helsinki (Becker and Grazia-Vieira 1971).

#### Bionomics.

Specimens mainly were collected by various types of light traps (UV light, mercury vapor light, black light) in or adjacent to dense forests. This species has never been collected by hand catching, sweeping, or beating vegetation during the day or night (JE Eger, pers. observ.). Adults have been collected in February, April–June, and August–December, with most in September and October (Figure 97) (Becker and Grazia-Vieira 1971; present paper).

#### Distribution

(Figs 99–100). Bolivia (Pirán 1956, as *Rh.grandicallosa*; this paper); Brazil: Amazonas, Rondônia (new country record); Ecuador (new country record); Peru (Arnold 2011; present paper); Venezuela (Becker and Grazia-Vieira1971, Grazia 1984).

### Rhyncholepta wheeleri sp. n.

Taxon classificationAnimaliaORDOFAMILIA

http://zoobank.org/CE334038-04E5-4140-8863-D1E18BE26578

Figs 8 , 25 , 40–41 , 52–53 , 64–65 , 71 , 77

#### Type locality.

Guyana, sources of Oronoque and New River (ca. 1°46'N 57°56'W).

#### Type material.

Holotype: ♂ (Figs 8, 25, 40–41, 52–53, 64–65, 71, 77), "BRITISH GUIANA: / Oronoque & New / River Heads. 1938. / H. Beddington. / B.M. 1938-346 [printed, white label] // ♂ [printed, white label] // HOLOTYPUS / *RHYNCHOLEPTA* / *WHEELERI* / sp. nov. / det. Kment, Eger, Rider 2017 [printed, red label]" (BMNH). The holotype is pinned through scutellum, antennomeres IIb to IV of both antennae, both middle legs and left hind leg missing; detached dissected abdomen + basi- (IIa) and distipedicellite (IIb) of one antenna, and genital capsule + abdominal segment VIII are glued on two separate pieces of card attached to the same pin.

#### Diagnosis.

Coloration, structure of head, thorax and pregenital abdomen, and vestiture as in other species of the genus (see redescription of *Rhyncholepta* above) except for the following characters.

*Apex of scutellum* with anteapical black V-shaped stripe on scutellum reduced to few black punctures anteapically near each lateral margin (Figure 25). Apical V-shaped callosity very narrow, concolorous with surrounding surface of scutellum, hardly apparent in the single specimen examined (Figure 25).

*Male genitalia.* Genital capsule in ventral view slightly constricted lateroapically, posterolateral angles obtusangulate, not prominent (Figs 40, 64); dorsal wall at base of posterolateral angles shallowly impressed (Figs 64, 71). Ventral rim in ventral view with wide M-shaped projection apically, shallow V-shaped incision medially; posterior hypandrial projections situated posterolaterally, short, pointed, directed ventrally (Figs 40–41); basal portions of posterior projections not visible in this view. Hypandrium in posterior view with three pairs of projections: posterior ones caudal, very short, acute but not spinose, directed ventrolaterad (Figs 52, 53: pp); lateral projections very narrow, directed posterolaterad, apices spinose, straight (Figs 52, 53: lp); anterior projections steeply sloping downwards, appearing narrowly parallel-sided, acutangulate apically, apices directed laterad (Figs 52, 53: ap). Anterior hypandrial projections in most exposed (dorso-posterolateral) view appearing roughly quadrangular, dorsal margins slightly concave, each apically with sharp spine strongly curved downwards (Figure 77: ap); bases of posterior and lateral projections widely separated, both projections parallel (Figure 77: a); posterior projection short, narrowing towards acute apex (Figure 77: pp); lateral projection long, spinous, nearly straight (Figure 77: lp). Anterior hypandrial projections in dorsal view parallel, each parallel-sided, abruptly sharpened apically, apices slightly divergent (Figs 64, 65: ap); posterior projections visible as acute angles (Figure 65: ppb); lateral projections directed laterad, narrowing to sharp spine, not curved apically (Figure 65: lp). *Phallus* not dissected in unique specimen available.

*Female.* Unknown.

*Measurements* of the holotype (see Table 1).

#### Differential diagnosis.

See above key.

#### Etymology.

The species is dedicated to Alfred G. Wheeler, Jr. (Department of Plant and Environmental Sciences, Clemson University, Clemson, South Carolina, USA), our friend and colleague, and excellent specialist in systematics and biology of Hemiptera. We feel it is appropriate that Tom Henry and Al Wheeler, long-time friends and co-authors of many papers, also share two species of the same genus.

#### Collecting circumstances.

Unknown.

#### Distribution

(Figure 99). Guyana (present paper).

## Supplementary Material

XML Treatment for Rhyncholepta

XML Treatment for Rhyncholepta grandicallosa grandicallosa

XML Treatment for Rhyncholepta grandicallosa centroamericana

XML Treatment for Rhyncholepta henryi

XML Treatment for Rhyncholepta meinanderi

XML Treatment for Rhyncholepta wheeleri
